# Quantitation and modeling of post-translational modifications in a therapeutic monoclonal antibody from single- and multiple-dose monkey pharmacokinetic studies using mass spectrometry

**DOI:** 10.1371/journal.pone.0223899

**Published:** 2019-10-16

**Authors:** Xiaobin Xu, Yu Huang, Hao Pan, Rosalynn Molden, Haibo Qiu, Thomas J. Daly, Ning Li

**Affiliations:** Regeneron Pharmaceuticals Inc., Tarrytown, New York, United States of America; Institut Pasteur, FRANCE

## Abstract

Post-translational modifications (PTMs) of therapeutic monoclonal antibodies (mAbs) are important product quality attributes (PQAs) that can potentially impact drug stability, safety, and efficacy. The PTMs of a mAb may change remarkably in the bloodstream after drug administration compared to *in vitro* conditions. Thus, monitoring *in vivo* PTM changes of mAbs helps evaluate the criticality of PQAs during the product risk assessment. In addition, quantitation of the subject exposures to PTM variants helps assess the impact of PTMs on the safety and efficacy of therapeutic mAbs. Here, we developed an immunocapture-liquid chromatography/mass spectrometry (LC/MS) method to quantify *in vivo* PTM changes a therapeutic mAb overtime in single- and multiple-dose monkey pharmacokinetic (PK) studies. We also built mathematical models to predict the *in vivo* serum concentrations of PQAs, the subject exposures to PQAs, and the relative abundance of PQAs in single- and multiple-dose regimens. The model predictions are in good agreement with the experimental results. The immunocapture-LC/MS method and mathematical models enable bioanalytical chemists to quantitatively assess the criticality of PQAs during drug development.

## Introduction

Therapeutic monoclonal antibodies (mAbs) produced in mammalian cells are heterogeneous as a result of post-translational modifications (PTMs). PTMs can occur during mAb production, purification, storage, and post-administration[1–4]. PTMs are a therapeutic mAb product quality attributes (PQAs). Controlling PQAs within predefined acceptance criteria is vital to the biopharmaceutical industry because it ensures consistent product quality and reduces potential impacts on drug safety and efficacy[5]. The modifications that occur during drug production and storage can be reliably monitored and controlled. However, additional modifications may occur after drug administration as a result of the different environments encountered by mAbs *in vivo* versus *in vitro*. Modifications of mAbs *in vivo* are usually challenging to monitor and less often studied[6]. Assessment and prediction of PTMs that can occur *in vivo* not only facilitate the understanding of quality attribute criticality for product risk assessment, but also help product development teams to engineer mAb drug candidates with enhanced *in vivo* stability.

To this end, a recent Food and Drug Administration (FDA) guidance for industry recommends sponsors to evaluate susceptibilities of therapeutic proteins to modifications that occur in the *in vivo* milieu[7]. As a result, *in vivo* behaviors of many PQAs, including deamidation[8–12], oxidation[10–12], glycation[13], glycosylation[14, 15, 11, 12], disulfide[16, 11], trisulfide[17], N-terminal pyroglutamate[10, 18, 11, 12], and C-terminal lysine removal[11, 19] have been recently investigated in animal or human samples. Affinity purification followed by liquid chromatography mass spectrometry (LC-MS) is often used to characterize PTMs of a mAb from *in vivo* samples. Affinity purification is used to extract a therapeutic mAb from serum samples to reduce the interference of the endogenous protein background prior to LC-MS analyses[11, 12]. For studies in animals, an anti-human antibody or the antibody target (antigen) can be used as a capture reagent. For human studies, the antibody target or an antibody that specifically recognizes the unique complementarity-determining region (CDR) of the therapeutic mAb can be used as a capture reagent. After affinity purification, LC-MS based peptide mapping is used to determine the relative abundance of PTM variants. Recently, *in vivo* PTM quantitation has been combined with pharmacokinetic (PK) models to evaluate the formation and elimination of PTM variants and subsequently quantitate the subject exposures to PQAs in single-dose regimens, helping establish the criticality of PQAs during product risk assessment[3, 11, 12]. However, modeling *in vivo* progression of PTMs and subject exposures to PQAs to assess the criticality of PQAs in multiple-dose regiments has not been attempted.

Here, we developed an affinity purification LC-MS to quantify PQAs of a therapeutic monoclonal antibody (MAB1), including deamidation, oxidation, N-terminal pyroglutamate formation, C-terminal lysine removal, and high mannose glycosylation, from a single-dose and a multiple-dose monkey PK studies. We built equations to establish the *in vivo* serum concentration of PQAs, the subject exposures to PQAs, and the *in vivo* relative abundance of PQAs in single- and multiple-dose regiments using three common PTMs (deamidation of two asparagine residues and N-terminal pyroglutamate formation) as the proof-of-concept modeling examples. The model predictions are in good agreement with experimental results from both single- and multiple-dose studies. This practice was conducted as a proof-of-concept work utilizing modeling approaches. Other PTMs were not selected for modeling due to either no change *in vivo* or extremely low abundance. The objective of this paper is to provide bioanalytical chemists a simplified modeling tool to quantitatively assess PQAs when advanced PK models are not available, such as a PK model incorporating both MAB1 and PQAs and describing the concentrations of each component concurrently. Thus, selection of the common PTMs as the proof-of-concept modeling examples allows readers to validate equations and apply the models on the PQAs on their own molecules. As a potential application example of the models, we used our models to evaluate the changes of the subject exposures to a hypothetical CDR deamidation in both single- and multiple-dose regimens to help assess the criticality of the PQAs of therapeutic mAbs during the product risk assessment.

## Materials and methods

### Materials

The human IgG4 monoclonal antibody (MAB1) and the anti-human antibody used in this study were produced by Regeneron Pharmaceuticals, Inc. (Tarrytown, NY). The mean half-life of MAB was approximately 11.5 days. Unless otherwise indicated, all reagents were purchased from Sigma-Aldrich (St. Louis, MO) or Thermo Fisher Scientific (Waltham, MA).

### Preclinical sample information

The preclinical serum samples were obtained from either single-dose or multiple-dose cynomolgus monkey PK studies. The studies complied with all applicable sections of the Final Rules of the Animal Welfare Act regulations (Code of Federal Regulations, Title 9), the *Public Health Service Policy on Humane Care and Use of Laboratory Animals* from the Office of Laboratory Animal Welfare, and the *Guide for the Care and Use of Laboratory Animals* from the National Research Council. The protocol and any amendments or procedures involving the care or use of animals in this study were reviewed and approved by the Testing Facility Institutional Animal Care and Use Committee before the initiation of such procedures. If an animal was determined to be in overt pain/distress, or appears moribund and was beyond the point where recovery appears reasonable, the animal was euthanized for humane reasons in accordance with the *American Veterinary Medical Association (AVMA) Guidelines on Euthanasia* and with the procedures outlined in the protocol. The housing conditions for animals on study are described as follows: Animals were housed in stainless steel cages equipped with a stainless steel mesh floor and an automatic watering valve. Primary enclosures were as specified in the USDA Animal Welfare Act (9 CFR, Parts 1, 2 and 3) and as described in the Guide for the Care and Use of Laboratory Animals. Each cage was clearly labeled with a cage label indicating study, group, animal and tattoo number, and sex. The targeted conditions for animal room environment are described as follows: Temperature: 64°F to 84°F; Humidity: 30%-70%; Light Cycle: 12 hours light and 12 hours dark; Ventilation: Ten or more air changes per hour with 100% fresh air (no-recirculation). The food conditions are described as follows: PMI Nutrition International Certified Primate Chow No. 5048 (25% protein) was provided daily, except during designated procedures. The chow was provided in amounts appropriate for the size and age of the animals. The feed was analyzed by the supplier for nutritional components and environmental contaminants. The diet was supplemented with fruit or vegetables at least 2–3 times weekly. No contaminants are known to be present in the certified diet at levels that would interfere with the results of this study. The water conditions are described as follows: Municipal tap water after treatment by reverse osmosis and ultraviolet irradiation was freely available to each animal via an automatic watering system (except during designated procedures). Periodic analysis of the water was performed. No contaminants were known to be present in the water at levels that would interfere with the results of this study. The animal enrichment is described as follows: Unless precluded for technical, scientific, or health reasons, animals were socialized to provide for psychological enrichment. Up to 3 females were commingled within dose groups after initial compatibility tests were completed. Animals were separated during designated procedures/activities. As a reward and means to promote operant conditioning and desired behavior, each animal may be offered a food treat following study-related procedures (e.g., dosing or sample collection) as deemed necessary throughout the duration of the study. The source of animals were Cynomolgus monkeys with China origin. Animals were observed twice daily. Additionally, animals were observed at each study blood collection time point. Body weights were recorded within 2 days prior to dosing on Day 1. MAB1 was administered to subjects intravenously (IV). For the single-dose study, animals were dosed at 10 mg/kg, and serum samples were collected at designated time points (pre-dose, 5-minute, 4-hour, 12-hour, 1-day, 3-day, 7-day, 14-day, 18-day, 30-day, 42-day, and 56-day) over the course of 8 weeks (56 days). For the multiple-dose study, animals were dosed at 3 mg/kg every 2 weeks (14 days), and serum samples were collected at designated time points (pre-dose, 1-hour, 4-hour, 1-day, 3-day, 7-day) within the first dosing interval as well as before and after each new dose (14-day, 28-day, 42-day, 56-day) over the course of 8 weeks (56 days). The serum samples were stored at -80°C until analyses. The MAB1 serum concentration at each collected time-point was measured using an enzyme-linked immunosorbent assay (ELISA). In brief, the MAB1 was captured on a microtiter plate coated with drug target. The MAB1 captured on the plate was detected using biotinylated mouse anti-human IgG4 monoclonal antibody, followed by NeutrAvidin conjugated to horseradish peroxidase (NeutraAvidin-HRP). A luminol-based substrate specific for peroxidase was then added to achieve a signal intensity that is proportional to the concentration of MAB1.

### Affinity purification of MAB1 from serum samples

MAB1 was purified from the collected monkey serum samples by affinity purification. In brief, a biotinylated anti-human antibody was conjugated to Dynabeads MyOne Streptavidin T1 magnetic beads (Invitrogen, Carlsbad, CA) at room temperature for 10 minutes. The conjugated beads were then incubated with serum samples at room temperature for 30 minutes. The beads were washed with HBS-EP buffer (GE Healthcare, Pittsburgh, PA), and then eluted with 0.1% formic acid (FA) and 50% acetonitrile. A Bioanalyzer (Agilent Technologies, Santa Clara, CA) was used to assess the recovery rate of MAB1 during the affinity purification. A known amount of 230 kDa high molecular-weight standard from Agilent (shown as “Upper Marker”) was run with each sample as an internal normalization control. The ratio of the MAB1 peak area to the Upper Marker peak area in each sample was calculated to correct for run-to-run variability. The recovery rate of MAB1 during the affinity purification was calculated using the following equation:

$$Recovery\;Rate=\frac{Peak\;Area\;Ratio\;of\;MAB1\;to\;Upper\;Marker\;in\;"Eluate"}{Peak\;Area\;Ratio\;of\;MAB1\;to\;Upper\;Marker\;in\;"MAB1\;Input"}\times 100\%$$ (1)

### Tryptic digestion

The purified MAB1 samples were dried down using a vacuum concentrator (LABCONCO, Kansas City, MO). The dried samples were re-suspended in 100 mM Tris-HCl containing 8 M urea and 10 mM Tris (2-carboxyethyl) phosphine hydrochloride (TCEP-HCl), and then incubated at 37°C for 30 minutes. The reduced cysteine residues were alkylated with 10 mM iodoacetamide at room temperature for 30 minutes in the dark. Following alkylation, the urea concentration was diluted to 1.25 M prior to digestion. Trypsin (Promega, Sunnyvale, CA) was added the samples at an enzyme: substrate ratio of 1:10 and incubated at 37°C for 4 hours. Digestion was terminated by addition of 20% formic acid. The digested samples were stored at -80°C until analysis.

### LC-MS/MS and data analysis

Peptides generated by trypsin digestion were separated using an Acquity UPLC CSH C18 1.7 μm, 2.1 mm × 150 mm column (Waters, Milford, MA) on an Acquity I-Class UPLC system (Waters, Milford, MA) coupled to a Q Exactive Plus mass spectrometer (Thermo Fisher Scientific, San Jose, CA). Mobile phase A was 0.1% FA in water and mobile phase B was 0.1% FA in acetonitrile. A gradient increasing from 2% mobile phase B to 30% mobile phase B over 56 min at a flow rate of 0.25 mL/min was used for peptide separation. The MS acquisition consisted of a full mass scan followed by tandem mass (MS/MS) scans of the top 5 highest intensity ions from each full scan. Peptide and PTM identification were determined by Byonic (version 2.16.11, Protein Metrics Inc., San Carlos, CA) and verified manually. To quantify relative abundance of PTMs, the extracted ion chromatograms, based on the m/z of the first isotope peak of both the modified peptide and native peptide, were generated and the extracted peak areas were integrated using Skyline-daily (version 4.1.1.18151, MacCoss Lab, University of Washington, WA) using a mass window of 5 ppm. The percentage of each PTM variant was calculated using the extracted ion chromatogram (EIC) peak area of the modified peptide relative to the sum of the peak areas of the modified and native peptides.

### Calculation of the subject exposure to a quality attribute in the single-dose PK study

Quantitative assessment of the subject exposure to a quality attribute in a single-dose PK study was described previously by Flynn *et al*.[3] We adopt this approach and briefly describe as follows. The MAB1 serum concentration-time equation can be described by a two-compartment model[20]:

$$C(t)=\mathrm{Dose}\times (C_{1}e^{‐\lambda_{1}t}+C_{2}e^{‐\lambda_{2}t})$$ (2),

where *C*(*t*) is the serum concentration of MAB1; *C*_*1*_, *C*_*2*_, *λ*_*1*_, and *λ*_*1*_ are hybrid constants.

1. To simplify for the readability, we adopt the format of

$$C(t)=Ae^{-\alpha t}+Be^{-\beta t}$$ (3),

where *A* and *B* are the zero-time intercepts (hybrid coefficients); *α* and *β* are the hybrid first-order constants, respectively. The equation was fitted to the ELISA-measured MAB1 serum concentrations at the collected time points to find the best-fit parameters. The fitting constrains were: C (0) must be greater than the first post-dose ELISA-measured MAB1 serum concentration; C (infinite) constant equal to 0; A and B must be greater than 0. R square was used to quantify goodness-of-fit and a R square greater than 0.95 was considered as a good fit.

The relative abundance of MAB1 with deamidation at a site-specific Asn can be described by a first-order kinetic equation:

$$P_{deam}(t)=1-(1-P_{0})∙e^{-k_{deam}t}$$ (4),

where *P*_*deam*_(*t*) is the percentage of the deamidation; *P*_0_ is the initial deamidation level on Day 0; *k*_*deam*_ is the deamidation rate constant.

The serum concentration of MAB1 with deamidation at a specific Asn site can be described as

$$C_{deam}(t)=C(t)∙P_{deam}(t)$$ (5),

where *C*_*deam*_(*t*) is the serum concentration of MAB1 with deamidation at a specific Asn site. The area under the curve (AUC) of *C*_*deam*_(*t*) represents the subject exposures to the quality attribute (i.e., MAB1 with deamidation at a specific Asn site. Equations describing *C*(*t*) and *P*_*deam*_(*t*) were solved by nonlinear regression using JMP (version 13.2.1, SAS, Cary, NC). *C*_*deam*_(*t*) was calculated using Excel (Microsoft, Redmond, WA) and plotted using JMP. The subject exposure, represented by AUC of *C*_*deam*_(*t*), was calculated using JMP.

### Calculation of the subject exposures to a quality attribute in the multiple-dose PK study

Modeling the subject exposure to a quality attribute in the multiple-dose *PK* study was mathematically simplified using the superposition principle as the linear accumulation of multiple single-doses. The serum concentration of MAB1 following each dose was described by a two-compartment model, discussed in the single-dose PK study above. The serum concentration of MAB1 immediately before the *m*^th^ dose can be described as follows:

$$C_{m,pre-dose}(m∙\tau )=C[(m-1)∙\tau ]+\cdots +C(1∙\tau )$$ (6),

where *m* is the number of doses and *τ* is the time interval between doses.

The serum concentration of MAB1 immediately after the *m*^th^ dose can be described as follows:

$$C_{m,post-dose}(m∙\tau )=C[(m-1)∙\tau ]+\cdots +C(1∙\tau )+C(0)$$ (7)

When *m* approaches infinity, the *C*_*m*,*pre*−*dose*_ and *C*_*m*,*after*−*dose*_ approach the steady-state concentrations, described as follows:

$$C_{\infty ,steady-state,pre-dose}=\sum_{i=1}^{\infty }(A∙e^{‐\alpha \;\tau i}+B∙e^{‐\beta \;\tau i})={\frac{A}{e^{\alpha \tau }-1}}^{}+{\frac{B}{e^{\beta \tau }-1}}^{}$$ (8)

$$C_{\infty ,steady-state,post-dose}=\sum_{i=0}^{\infty }(A∙e^{‐\alpha \;\tau i}+B∙e^{‐\;\beta \;\tau i})={\frac{Ae^{\alpha \;\tau }}{e^{\alpha \;\tau }-1}}^{}+{\frac{Be^{\beta \;\tau }}{e^{\beta \;\tau }-1}}^{}$$ (9)

The serum concentration of MAB1 with deamidation at a specific Asn site immediately before the *m*^th^ dose can be described as follows:

$$C_{deam,m,pre-dose}(m∙\tau ){=P}_{deam}[(m-1)∙\tau ]C[(m-1)∙\tau ]+\cdots +P_{deam}(1∙\tau )C(1∙\tau )$$ (10)

The serum concentration of MAB1 with deamidation at a specific Asn site determined immediately following the *m*^th^ dose can be described as follows:

$$C_{deam,m,post-dose}(m∙\tau )=P_{deam}[(m-1)∙\tau ]C[(m-1)∙\tau ]+\cdots +P_{deam}(1∙\tau )C(1∙\tau )+P_{deam}(0)C(0)$$ (11),

where *P*_*deam*_(*t*) is the first-order reaction equation, discussed in the single-dose PK study above.

Thus, the relative abundance of MAB1 with deamidation at a specific Asn site immediately before the *m*^*th*^ dose can be described as follows:

$$P_{deam,m,pre-dose}(m∙\tau )=\frac{C_{deam,m,pre-dose}(m∙\tau )}{C_{m,pre-dose}(m∙\tau )}$$ (12)

The relative abundance of MAB1 with deamidation at a specific Asn site immediately following the *m*^*th*^ dose can be described as follows:

$$P_{deam,m,post-dose}(m∙\tau )=\frac{C_{deam,m,post-dose}(m∙\tau )}{C_{m,post-dose}(m∙\tau )}$$ (13)

When *m* approaches infinity, the *P*_*deam*,*m*,*pre*−*dose*_ and *P*_*deam*,*m*,*after*−*dose*_ approach the steady-state levels, described as follows:

$$P_{deam,\infty ,steady-state,pre-dose}=\frac{1}{C_{\infty ,pre-dose}}\sum_{i=1}^{\infty }P_{deam}(i)∙C(i)=\frac{1}{C_{\infty ,pre-dose}}\sum_{i=1}^{\infty }\left[1-(1-P_{0})e^{-k\tau i}\right](A∙e^{‐\alpha \tau i}+B∙e^{‐\beta \tau i})$$ (14)

$$P_{deam,\infty ,steady-state,post-dose}=\frac{P_{deam,\infty ,pre-dose}∙C_{\infty ,pre-dose}+P_{deam}(0)∙C(0)}{C_{\infty ,post-dose}}$$ (15)

Since the calculation of the summation terms converges, the pre-dose levels of deamidation at the steady state can be simplified as follows:

$$P_{deam,\infty ,steady-state,pre-dose}=\frac{A(\frac{1}{e^{\alpha \tau }-1}-\frac{1-P_{0}}{e^{(\alpha +k)\tau }-1})+B({\frac{1}{e^{\alpha \tau }-1}}^{}-\frac{1-P_{0}}{e^{(\beta +k)\tau }-1})}{{\frac{A}{e^{\tau \;\alpha }-1}}^{}+{\frac{B}{e^{\tau \;\beta }-1}}^{}}$$ (16)

Therefore, the post-dose level of deamidation at the steady state can be simplified as follows:

$$P_{deam,\infty ,steady-state,post-dose}=\frac{\left[A(\frac{1}{e^{\alpha \;\tau }-1}-\frac{1-P_{0}}{e^{(\alpha +k)\tau }-1})+B({\frac{1}{e^{\beta \tau }-1}}^{}-\frac{1-P_{0}}{e^{(\beta +k)\tau }-1})]+P_{0}(A+B\right)}{{\frac{{Ae}^{\alpha \;\tau }}{e^{\alpha \;\tau }-1}}^{}+{\frac{Be^{\beta \;\tau }}{e^{\beta \;\tau }-1}}^{}}$$ (17)

The equation constants *A*, *B*, *α*, *β*, and *k* were solved by fitting the equation to the experimental results within the first dosing interval. The simulated and predicted values were calculated using Excel and then plotted using JMP. The subject exposure (*i*.*e*., AUC) was calculated using JMP.

## Results

### Quantitation of MAB1 PQAs in vivo using affinity purification LC-MS

The *in vivo* behaviors of MAB1 with site-specific PTMs were assessed in both single- and multiple-dose monkey PK studies.

**Fig 1:**
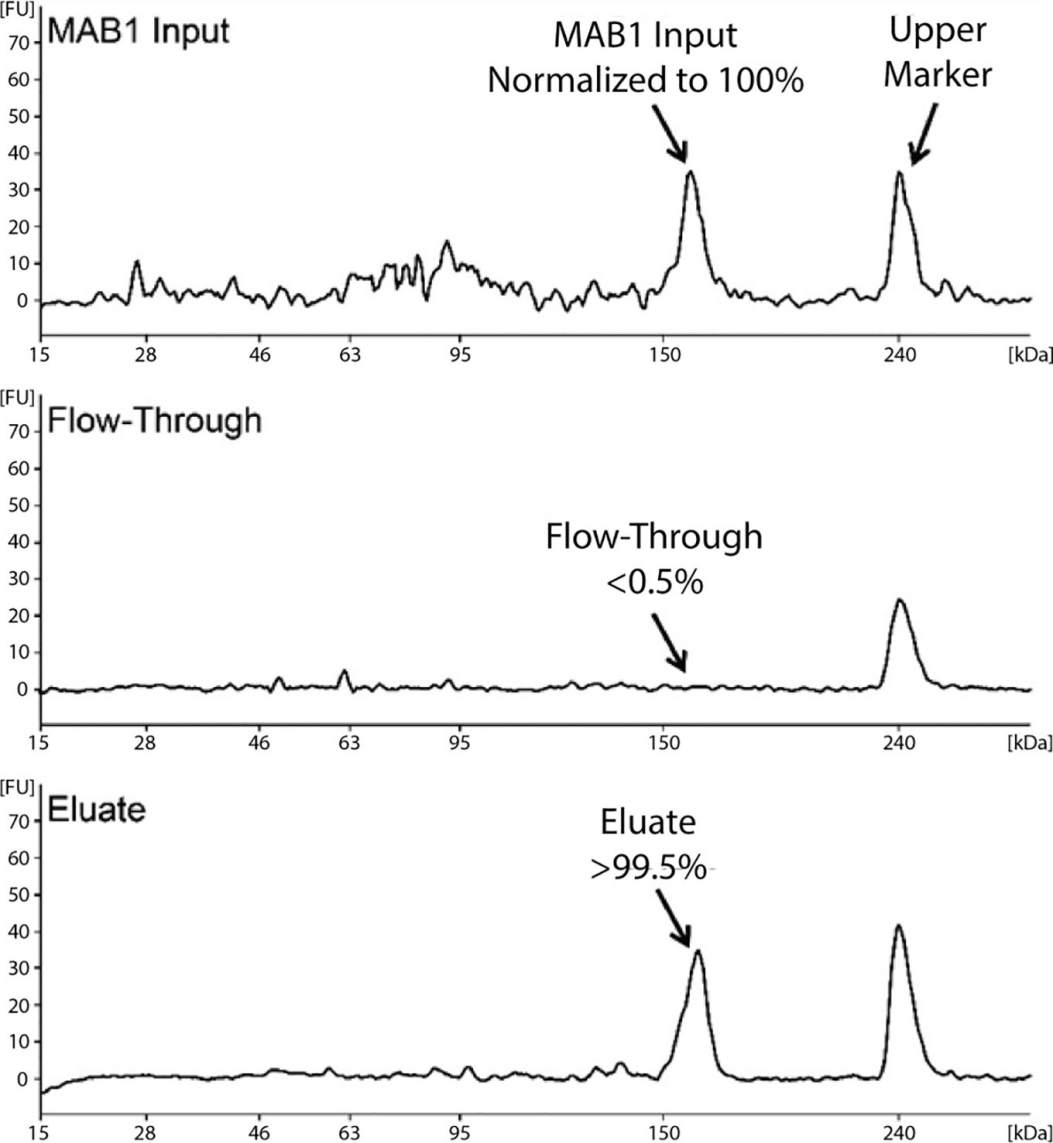
The recovery of the affinity purification of MAB1. The recovery rate of the affinity purification of MAB1 was >99.5% while <0.5% total MAB1 was detected in the flow-through. The calculation of percent recovery rate was described in the Materials and Methods section. A known amount of high molecular-weight standard (shown as “Upper Marker”) was run with each sample as an internal normalization control. The ratio of the MAB1 peak area to the Upper Marker peak area in each sample was calculated to correct for run-to-run variability.

A biotinylated anti-human Fc (Fragment Crystallizable region) antibody, conjugated to streptavidin magnetic beads, was used to extract MAB1 from the monkey serum samples. The affinity purification recovery of MAB1 was > 99.5% (Fig 1), demonstrating that the affinity purification can completely extract both wild-type MAB1 and MAB1 PTM variants without bias, ensuring accurate quantitation of the relative abundance of each PTM.

**Fig 2:**
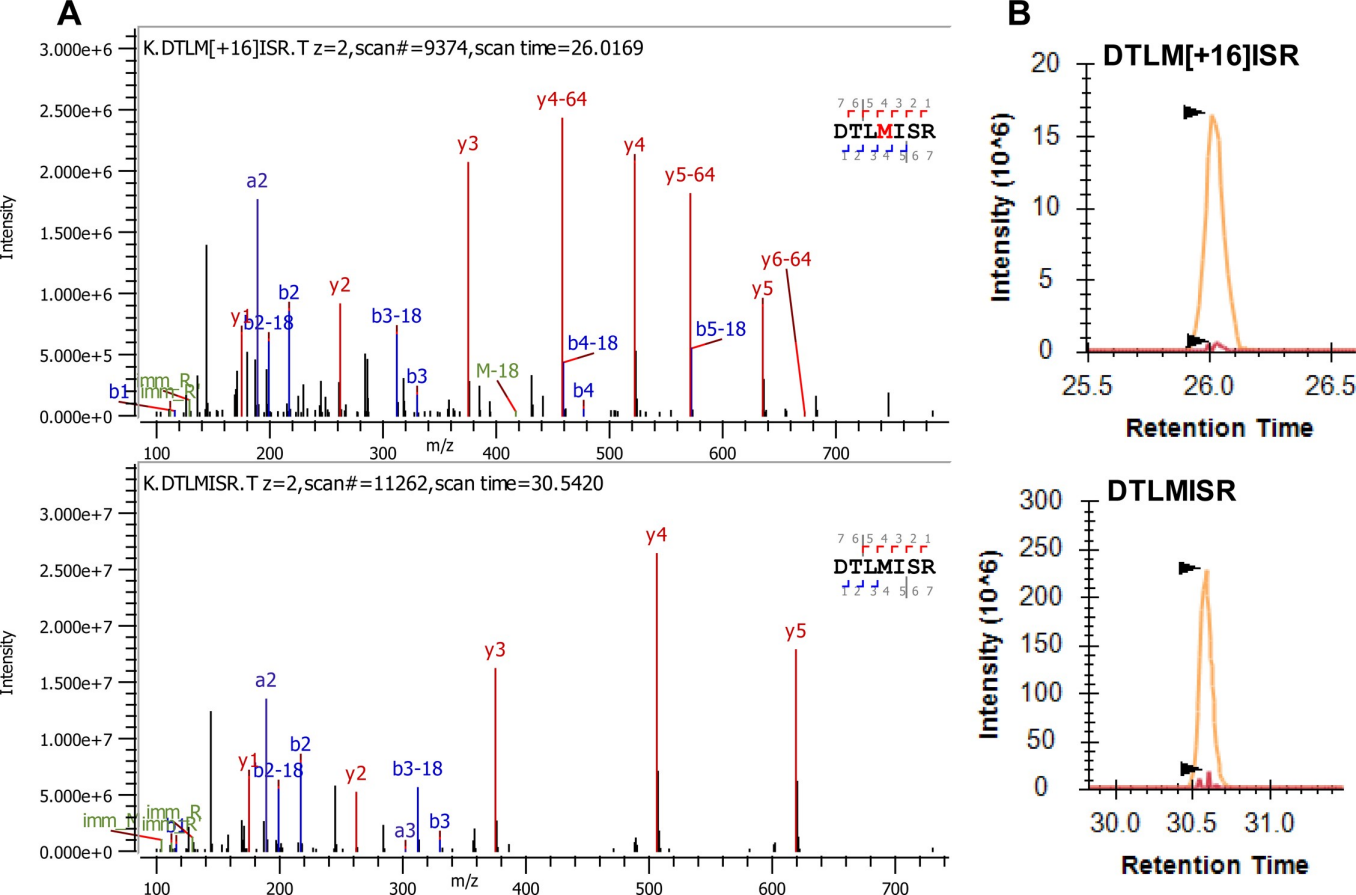
**Example MS/MS spectra of peptide identification (A) and example extracted ion chromatograms for peptide peak integration and PTM quantitation (B)**. **(A)** The MS/MS spectrum of an example Met oxidized (Met site 1) peptide, DTLMISR (top panel) and the MS/MS spectrum of the example wild-type peptide, DTLMISR (bottom panel). **(B)** Extracted ion chromatograph of the Met oxidized peptide, DTLMISR (top panel) and the wild-type peptide, DTLMISR (bottom panel).

Fig 2A shows example tandem MS spectra of the identification of a wildtype Fc tryptic peptide “DTLMISR” (bottom panel) and the corresponding methionine (Met Site 1) oxidized peptide (upper panel). Fig 2B shows the extracted ion chromatograph peak areas of the wildtype peptide (bottom panel) and the corresponding Met oxidized peptide (upper panel). Tandem spectra of other PTMs were shown in the Supporting information as follows: Oxidation of Met site 2 (S1 Fig) and site 3 (S2 Fig), Deamidation of Asn site 1 (S3 Fig), Asn site 2 (S4 Fig) and Asn site 3 (S5 Fig), N-terminal pyroglutamate formation (S6 Fig), C-terminal Lysine removal (S7 Fig), and mannose 5 (S8 Fig).

**Fig 3:**
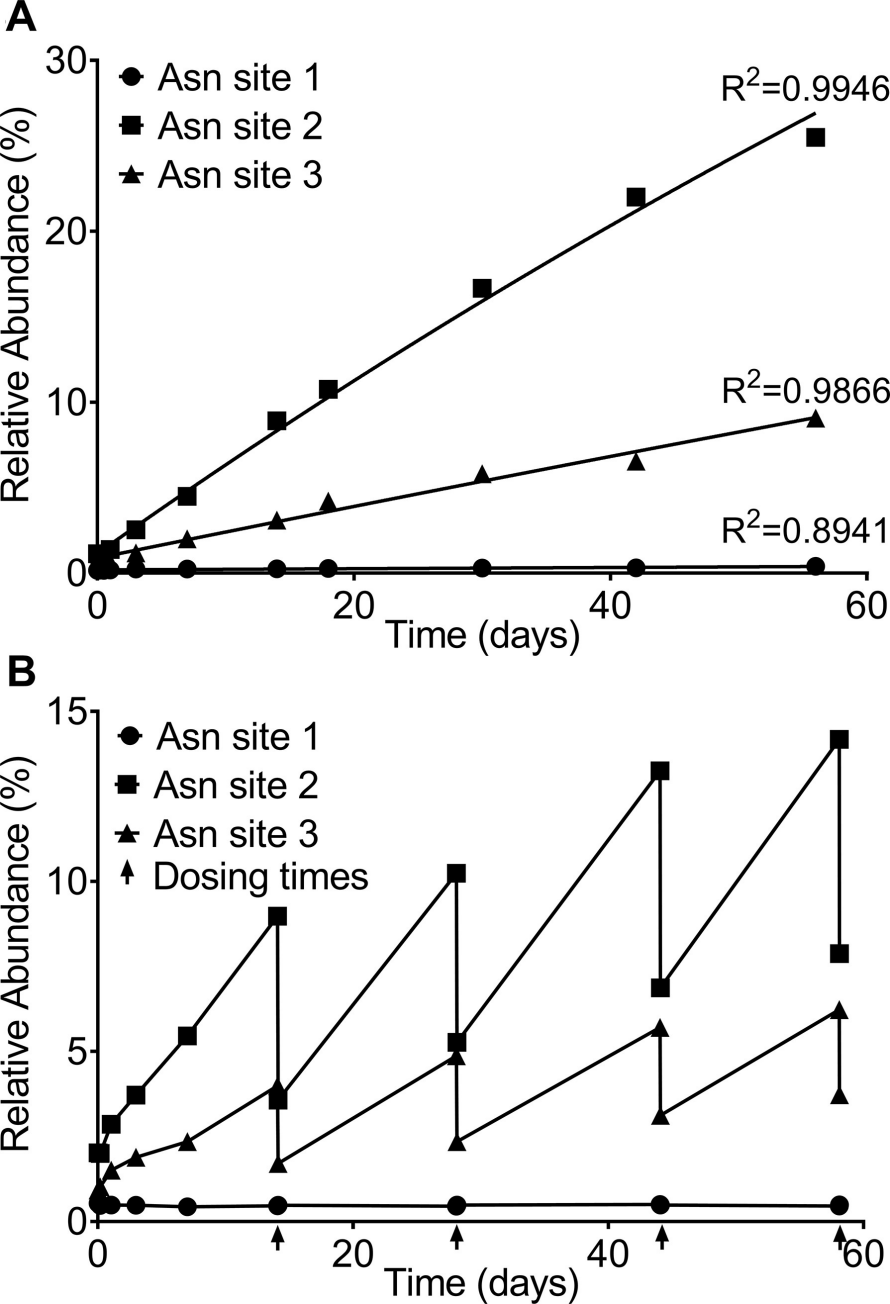
**The relative abundance of deamidation at each of the three Asn sites in the Fc region of MAB1 from the single-dose PK study (A) and the multiple-dose PK study (B)**. **(A)** In the single-dose study, the relative abundance of deamidation at each of the three Asn sites increased over time at different rates following the first-order kinetic equation: $P_{deam}(t)=1-(1-P_{0})∙e^{-k_{deam}t}$. Using non-linear regression, the *in vivo* deamidation rate constants at Asn site 1, 2, and 3 were determined to be 0.003523% day-1, 0.5394% day-1, and 0.1546% day-1, respectively. **(B)** In the multiple-dose study, the relative abundance of deamidation at each of the three Asn sites increased over time during each dosing interval following the first-order kinetic equation, but decreased sharply following each subsequent dose due to dilution with newly administrated unmodified MAB1, exhibiting an upward trending saw-tooth pattern. Each dosing time is indicated with an arrow “↑”.

Deamidation of asparagine (Asn, N) is a common PTM in mAbs[21]. An Asn residue followed by glycine (Gly, G), serine (Ser, S), or asparagine within the primary amino acid sequence is particularly susceptible to deamidation[22]. In this work, three Asn deamidation sites, “NG” (Asn site 1) in the CH2 domain, “NG” (Asn site 2) and “NN” (Asn site 3) in the CH3 domain were monitored. In the single-dose PK study, the relative abundance of deamidation at Asn site 1 remained nearly unchanged at a low level of ~0.2% over 56 days (Fig 3A). The relative abundance of deamidation at Asn site 2 and 3 increased from ~1.0% to 25.5% and 9.1%, respectively, over 56 days (Fig 3A). The deamidation rate largely depends on the primary amino acid sequence, protein structure, solvent accessibility, pH, temperature, and buffer composition[23]. The “NG” and “NN” sites in the CH3 domain are more exposed to solvent, and therefore more susceptible to deamidation than the “NG” site in the CH2 domain[24].

Deamidation is a first-order reaction[25]. The deamidation rate equation can be described as a first-order rate equation: $P_{deam}(t)=1-(1-P_{0})∙e^{-k_{deam}t}$, as decribed in the Materials and Method section. The equation was non-linearly fitted to the LC-MS measured deamidation levels in the single dose study to determine the best-fit parameters (Fig 3A and Table 1). The *in vivo* deamidation rate constants of MAB1 at Asn site 1, 2, and 3 were determined to be 0.003523% day^-1^, 0.5394% day^-1^, and 0.1546% day^-1^, respectively, with a R^2^ of 0.9946, 0.9866, and 0.8941, respectively (Table 1).

**Table 1:** The best-fit parameter values in the modification rate equations from the single-dose PK study.

Deamidation Site	*P*_0_ (%)	*k*_deam_ (day^-1^)	Standard Error of fitted *P*_0_	Standard Error of fitted *k*_deam_	R^2^
Asn site 1	0.1918	0.003523%	0.00971	4.044e-006	0.8941
Asn site 2	1.143	0.5394%	0.2758	0.0001361	0.9946
Asn site 3	0.8848	0.1546%	0.1389	6.09e-005	0.9866
N-terminal pyroglutamate	0.6868	0.2201%	0.1169	5.22e-005	0.9970

Where *P*_0_ is the initial deamidation level on Day 0; *k*_*deam*_ is the deamidation rate constant.

In the multiple-dose PK study, the relative abundance of deamidation at Asn site 1 remained nearly unchanged at a low level of ~0.5% following 5 biweekly doses over 56 days (Fig 3B). The relative abundance of deamidation at Asn site 2 and 3 accumulated within each dosing interval but dropped sharply after each new dose because of dilution with newly administrated non-deamidated MAB1 (Fig 3B). The pre-dose and post-dose levels of deamidation gradually increased following each dose due to the accumulation of deamidated MAB1. The pre-dose level of deamidation at Asn site 2 prior to the 5^th^ dose on Day 56 increased to 14.2% while the post-dose level reached 7.9% (Fig 3B). The pre-dose level of deamidation at Asn site 3 prior to the 5^th^ dose on Day 56 increased to 6.2% while the post-dose level reached 3.6% (Fig 3B). As a result, the relative abundance of deamidation at Asn site 2 and 3 exhibited a saw-tooth pattern with an increasing trend over time (Fig 3B). Fig 3B also suggests that both pre-dose and post-dose levels of deamidation will approach to a steady-state after a finite number of doses. To predict the steady-state levels of deamination, we built a 2-compartment model for MAB1 concentrations that will be discussed in the modeling sections of this paper.

Met oxidation is another common PTM in mAbs[10, 18, 11, 12]. Met oxidation in the Fc region has been shown to impact the Fc receptor binding[26]. In this work, we evaluated three common Met oxidation sites in the Fc region, Met site 1 in the CH2 domain as well as Met site 2 and 3 in the CH3 domain. In the single-dose study, the relative abundance of oxidation at Met site 1, 2, and 3 were relatively unchanged over 56 days at a level of ~8.0%, ~2.0%, and ~5.0%, respectively (S9 Fig). In the multiple-dose study, the level of oxidation at Met site 1 decreased slightly during each dosing interval and increased slightly after each dose, exhibiting a saw-tooth pattern that fluctuates between ~7.0% and ~9.0%. The levels of oxidation at Met site 2 and 3 remained stable at a level of ~1.0% and 4.0%, respectively, in the multiple-dose study (S9 Fig).

The N-terminal glutamine and glutamate of mAbs are prone to form pyroglutamate through chemical or enzymatic cyclization[10, 18, 11, 12]. The conversion of glutamine occurs much faster than that of glutamate. The N-terminal cyclized protein is resistant to digestion by aminopeptidases, thereby inhibiting degradation. Although glutaminyl cyclase, an enzyme that catalyzes this conversion, is found in human blood[27], the formation of pyroglutamate from glutamate in mAbs *in vivo* is predominantly a pH-dependent non-enzymatic first-order reaction, as it bears the same rate constant in PBS *in vitro*[18, 10]. In the single-dose study, the level of N-terminal pyroglutamate increased from 0.7% to 11.7% over 56 days (S10 Fig). The *in vivo* N-terminal pyroglutamate formation rate was determined to be 0.2201% day^-1^ with a R^2^ of 0.9970 using non-linear fitting to the LC-MS measured N-terminal pyroglutamate levels. In the multiple-dose study, N-terminal pyroglutamate formation resembled a saw-tooth pattern similar to Asn deamidation (S10 Fig). The level of pyroglutamate increased during each dosing interval but reduced sharply after each new dose as a result of dilution with newly administrated unmodified MAB1. Prior to the 5^th^ dose on Day 56, the level of pyroglutamate raised to 7.6% and then dipped to 4.2% following the 5^th^ dose (S10 Fig).

The C-terminal lysine residue in the heavy chain of mAbs is susceptible to removal by basic carboxypeptidase during protein expression[28]. Partial removal of the C-terminal lysine leads to charge heterogeneity. The relative abundance of MAB1 with C-terminal lysine before administration was 2.3%. In both single and multiple-dose studies, the C-terminal lysine was rapidly removed within a single day following each dose (S11 Fig). These observations are consistent with a previously reported study[19].

N-linked glycosylation in the Fc region is a commonly monitored PQA of a mAb. The predominant structures of N-linked glycoforms on Chinese Hamster Ovary (CHO) expressed mAbs are typically composed of a core bi-antennary pentasaccharide structure with one or more additional monosaccharides attached. In addition to the complex bi-antennary oligosaccharide structures, high-mannose glycoforms (e.g., Mannose 5) can also be observed on CHO-expressed antibodies. In the single-dose study, the relative abundances of the major glycoforms (fucosylated complex bi-antennary) remained unchanged over 56 days (S13 Fig). However, the relative abundance of Mannose 5 glycoform decreased from 0.5% to undetectable within 6 weeks (S12 Fig). This finding agrees with the faster serum clearance of high-mannose glycoforms due to a high-mannose receptor-mediated clearance pathway[14, 15]. In the multiple-dose study, the level of Mannose 5 decreased during each dosing intervals and evaluated immediately following each new dose because of blending with newly administrated MAB1 with a higher level of Mannose 5, exhibiting a saw-tooth pattern (S12 Fig). Prior to administration of the 5^th^ dose, the level of Mannose 5 decreased to 0.1%. After administration of the 5^th^ dose, the level of Mannose 5 increased to 0.4%.

### Modeling the subject exposure to the MAB1 PQAs in the single-dose study

Quantitative assessment of the subject exposures to mAb quality attributes helps evaluate the impact of quality attributes on drug safety and efficacy. The *in vivo* serum concentration of a mAb with a certain PTM can be described as *C*_*PTM*_(*t*) = *C*(*t*)∙*P*(*t*) [29], as described in the Materials and Method section. The area under the curve (AUC) of *C*_*PTM*_(*t*) corresponds to the subject exposure to the mAb with the PTM[3]. In this work, we used deamidation at Asn site 2 and 3 as well as the N-terminal pyroglutamate formation as examples to demonstrate the modeling of the subject exposure to PQAs in the single-dose study. The modeling was not performed on other PTMs discussed in this paper because Met oxidation was not changed *in vivo* (S9 Fig); the C-terminal lysine was rapidly removed within hours after administration (S11 Fig); the levels of Mannose 5 and deamidation at Asn site 1 were extremely low (less than 0.5%, Fig 3 and S12 Fig), making the modeling of these PTMs may not be practically meaningful.

**Fig 4:**
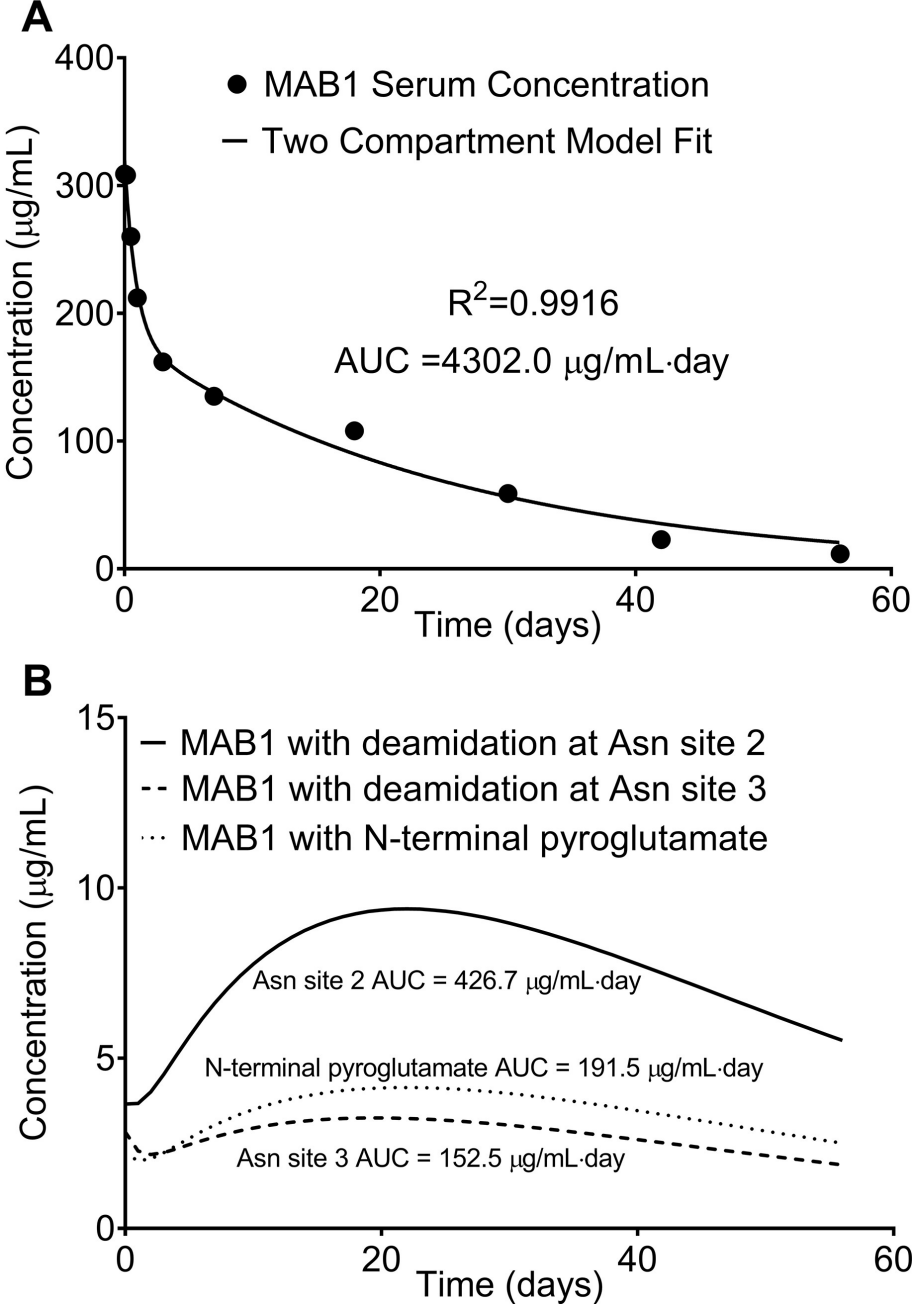
**The subject exposure to total MAB1 from the single-dose PK study (A) and the subject exposure to MAB1 with a PQA from the single-dose study (B)**. **(A)** The MAB1 serum concentration-time curve (black line) described by the two-compartment pharmacokinetic model equation as ***C*(*t*) = *Ae***^**−*αt***^**+*Be***^**−*βt***^ was fitted to the ELISA measured MAB1 serum concentrations (black dots). The subject exposure to total MAB1 over the course of 56 days, represented by the AUC of ***C*(*t*)** was determined as 4302.0 μg/mL∙day. **(B)** The serum concentration-time curve of MAB1 with deamidation is described as ***C***_***PQA***_**(*t*) = *C*(*t*)∙*P***_***PQA***_**(*t*)**, The AUC of the ***C***_***PQA***_**(*t*)** curve corresponds to the subject exposure to MAB1 with the PQA. The subject exposure to MAB1 with deamidation at Asn site 2 over 56 days was determined as 426.7 μg/mL∙day (solid line). The subject exposure to MAB1 with deamidation at Asn site 3 over 56 days was determined as 152.5 μg/mL∙day (hyphenated line). The subject exposure to MAB1 with N-terminal pyroglutamate over 56 days was determined as 191.5 μg/mL∙day (dot line).

The MAB1 serum concentration-time equation was described by a two-compartment pharmacokinetic model as[29] *C*(*t*) = *Ae*^−*αt*^+*Be*^−*βt*^, where A and B are the hybrid coefficients; α and β are the hybrid first-order constants. The experimental MAB1 serum concentrations from the single-dose PK study were determined using an ELISA assay. The modeling equation was solved by non-linear fitting to the ELISA measured MAB1 serum concentrations with a R^2^ of 0.9916 (Fig 4A). The best-fit values of the parameters in the modeling equation are shown in Table 2. The subject exposure to the total MAB1 over 56 days, represented by the AUC of *C*(*t*), was 4302.0 μg/mL∙day (Fig 4A).

**Table 2:** The best-fit parameters values in the MAB1 serum concentration-time equations from the single-dose PK study and the first dose interval of the multiple-dose PK study.

Study	The single-dose study	The first dose of multiple-dose study
*A*	139.0925	57.8676
*B*	180.8075	25.3133
*α*	1.1227	0.0413
*β*	0.0389	0.9286
Standard Error of fitted of A	15.4245	9.9061
Standard Error of fitted of B	17.3316	9.7499
Standard Error of fitted *α*	0.3262	0.0185
Standard Error of fitted β	0.0058	0.7592
R^2^	0.9916	0.9818

The rate equation of deamidation at Asn site 2 was determined in the previous section above as *P*_*deam*−*Asn*2_(*t*) = 1−(1−0.01143)∙*e*^−0.005394*t*^. The serum concentration-time equation of MAB1 with deamidation at Asn site 2, *C*_*deam*−*Asn*2_(*t*) = *C*(*t*)∙*P*_*deam*−*Asn*2_(*t*), can therefore be solved by multiplication of *C*(*t*) and *P*_*deam*−*Asn*2_(*t*). The curve of *C*_*deam*−*Asn*2_(*t*) was plotted in Fig 4B. The subject exposure to the MAB1 with deamidation at Asn site 2 over 56 days, represented by the AUC of *C*_*deam*_(*t*), was calculated to be 426.7 μg/mL∙day (Fig 4B), consisting of 9.9% of the subject exposure to total MAB1 over 56 days.

Similarly, the rate equation of deamidation at Asn site 3 was determined in the previous section above as *P*_*deam*−*Asn*3_(*t*) = 1−(1−0.008848)∙*e*^−0.001546*t*^. The serum concentration-time equation of MAB1 with deamidation at Asn site 3, *C*_*deam*−*Asn*3_(*t*) = *C*(*t*)∙*P*_*deam*−*Asn*3_(*t*), can therefore be solved by multiplication of *C*(*t*) and *P*_*deam*−*Asn*3_(*t*). The curve of *C*_*deam*−*Asn*3_(*t*) was plotted in Fig 4B. The subject exposure to the MAB1 with deamidation at Asn site 3 over 56 days, represented by the AUC of *C*_*deam*−*Asn*3_(*t*), was calculated to be 152.5 μg/mL∙day (Fig 4B), consisting of 3.5% of the subject exposure to total MAB1 over 56 days.

The rate equation of N-terminal pyroglutamate formation was determined in the previous section above as *P*_*PyroE*_(*t*) = 1−(1−0.006868)∙*e*^−0.002201*t*^. The serum concentration-time equation of MAB1 with N-terminal pyroglutmate, *C*_*PyroE*_(*t*) = *C*(*t*)∙*P*_*PyroE*_(*t*), can therefore be solved by multiplication of *C*(*t*) and *P*_*PyroE*_(*t*). The curve of *C*_*PyroE*_(*t*) was plotted in Fig 4B. The subject exposure to the MAB1 with N-terminal pyroglutmate over 56 days, represented by the AUC of *C*_*PyroE*_(*t*), was 191.5 μg/mL∙day (Fig 4B), consisting of 4.5% of the subject exposure to total MAB1 over 56 days.

### Modeling the in vivo serum concentrations of the MAB1 PQAs in the multiple-dose study

Modeling the subject exposure to a quality attribute from the multiple-dose PK study was mathematically simplified using the superposition principle, as described in the Materials and Methods section. A multiple-dose study is considered as the linear accumulation of a series of single-doses. The MAB1 serum concentration-time equation following each dose was described by a two-compartment model, as described in the single-dose PK study above. The ELISA measured MAB1 serum concentrations at the collected time points within the first dosing interval were fitted to the two-compartment model equation, *C*(*t*) = *Ae*^−*αt*^+*Be*^−*βt*^, to solve the equation parameters. The best-fit values of the parameters in the modeling equation are shown in Table 2. These parameters were then used in the equations to calculate mAb concentrations, PQA concentrations, and subject exposures to PQAs in the multiple-dose PK study.

**Fig 5:**
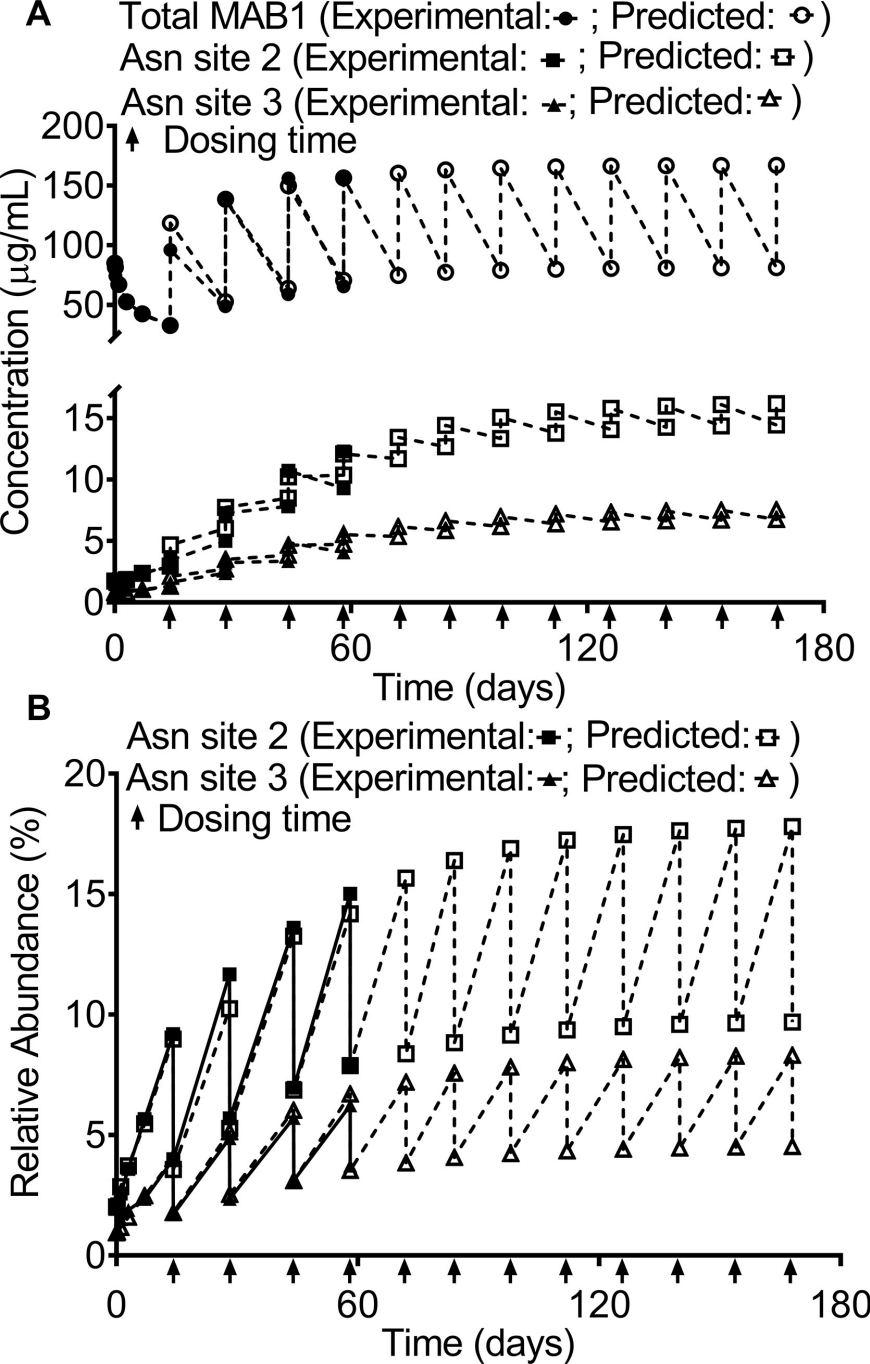
**Model predictions and experiment measurements of the serum concentration of total MAB1 and MAB1 with deamidation at Asn site 2 or Asn site 3 from the multiple-dose PK study (A) and model predictions and experimental measurements of the relative abundances of deamidation at Asn site 2 or Asn site 3 (B)**. **(A)** The predicted pre-dose and post-dose serum concentrations of total MAB1 and MAB1 with deamidation at Asn site 2 or Asn site 3 are in good agreement with the experimental measurements. The pre-dose and post-dose concentrations of total MAB1 and MAB1 with deamidation at Asn site 2 or 3 approach the steady-state levels following an extended period of dosing. **(B)** The predicted levels are in good agreement with the experimental values. The pre-dose and post-dose relative abundances of deamidation at Asn site 2 or Asn site 3 approach the steady-state levels following an extended period of dosing. Each dosing time is indicated with an arrow “↑”.

The pre-dose and post-dose serum concentrations of MAB1 at any given dose in the multiple-dose study can be predicted using Eqs 6 and 7, respectively. For example, the predicted pre-dose and post-dose concentrations of MAB1 at the 5^th^ dose on Day 56 are 70.6 μg/mL and 156.4 μg/mL, respectively. The predicted values agree with the ELISA determined pre-dose and post-dose concentrations of MAB1 at the 5^th^ dose on Day 56, which were 65.5 μg/mL and 156.0 μg/mL, respectively. All other predicted pre-dose and post-dose MAB1 serum concentrations (Fig 5A, hollow circles) are in good agreement with the ELISA measured MAB1 serum concentrations (Fig 5A, solid dots). The pre-dose and post-dose concentrations of MAB1 will approach the steady-state concentrations (i.e., the plateau concentrations) following a finite number of doses (Fig 5A, hollow circles). Using Eqs 8 and 9, the pre-dose and post-dose steady-state serum concentrations of MAB1 can be predicted to be 81.4 μg/mL and 167.2 μg/mL, respectively. At the 10^th^ dose (18 weeks, 126 days), the predicted pre-dose and post-dose concentrations of MAB1 are 80.5 μg/mL and 166.3 μg/mL, respectively, close to the steady-state concentrations.

We used deamidation at Asn site 2 and 3 as well as the N-terminal pyroglutamate formation as modeling examples in the multiple-dose study. For the same reasons in the single-dose study, the modeling was not performed on other PTMs discussed in this paper because Met oxidation was not changed *in vivo* (S9 Fig); the C-terminal lysine was rapidly removed within hours after administration (S11 Fig); the levels of deamidation at Asn site 1 and Mannose 5 were extremely low (less than 0.5%, Fig 3 and S12 Fig).

The pre-dose and post-dose serum concentrations of MAB1 possessing a specific attribute at any given dose in the multiple-dose study can be predicted using Eqs 10 and 11, respectively. The predicted pre-dose and post-dose concentrations of MAB1 with deamidation at Asn site 2 (Fig 5A, hollow squares) are consistent with the experimental results (Fig 5A, solid squares). The experimental concentrations of MAB1 with deamidation at Asn site 2 were calculated using the multiplication of the ELISA measured MAB1 concentration and the LC-MS measured percentage of deamidation at Asn site 2. The pre-dose and post-dose concentrations of MAB1 with deamidation at Asn site 2 will approach the steady-state concentrations after a finite number of doses (Fig 5A, hollow squares). Using Eqs 8, 9, 16 and 17, the pre-dose and post-dose steady-state concentration of MAB1 with deamidation at Asn site 2 are 14.6 μg/mL and 16.2 μg/mL, respectively. At the 12^th^ dose (22 weeks, 154 days), the predicted pre-dose and post-dose concentrations of MAB1 with deamidation at Asn site 2 are 14.4 μg/mL and 16.1 μg/mL, respectively, close to the steady-state concentrations.

Similarly, the predicted pre-dose and post-dose concentrations of MAB1 with deamidation at Asn site 3 (Fig 5A, hollow triangles) are consistent with the experimental results (Fig 5A, solid triangles). The pre-dose and post-dose concentrations of MAB1 with deamidation at Asn site 3 will also approach the steady-state concentrations after a finite number of doses (Fig 5A, hollow squares). Using Eqs 8, 9, 16 and 17, the pre-dose and post-dose steady-state concentration of MAB1 with deamidation at Asn site 3 are 6.8 μg/mL and 7.6 μg/mL, respectively. At the 12^th^ dose (22 weeks, 154 days), the predicted pre-dose and post-dose concentrations of MAB1 with deamidation at Asn site 3 are 6.7 μg/mL and 7.5 μg/mL, respectively, close to the steady-state concentrations.

**Fig 6:**
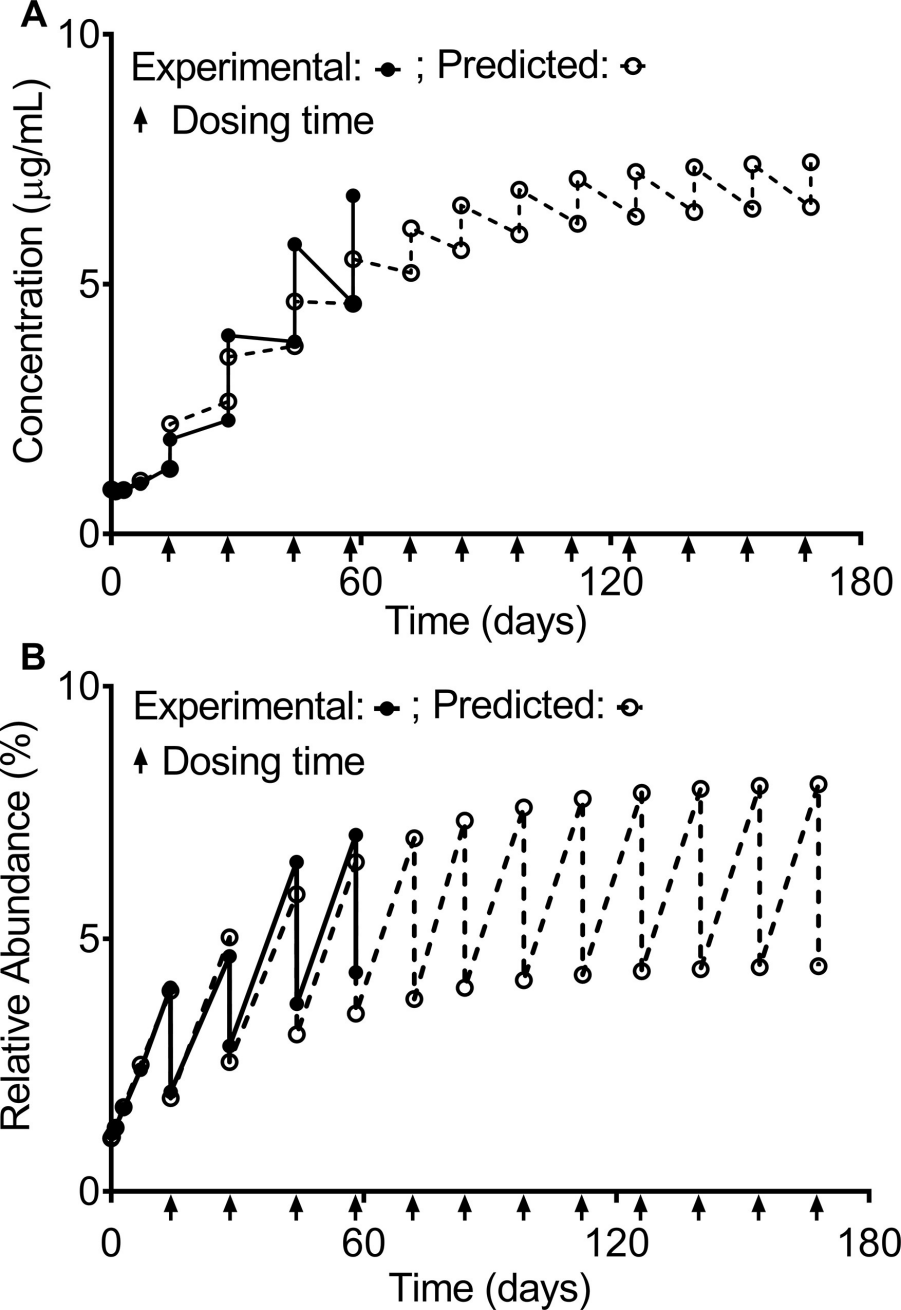
**Model predictions and experiment measurements of the serum concentration of MAB1 with N-terminal pyroglutamate from the multiple-dose PK study (A) and model predictions and experimental measurements of the relative abundances of N-terminal pyroglutamate (B)**. **(A)** The predicted pre-dose and post-dose serum concentrations of MAB1 with N-terminal pyroglutamate are in good agreement with the experimental measurements. The pre-dose and post-dose concentrations of MAB1 with N-terminal pyroglutamate approach the steady-state levels following an extended period of dosing. **(B)** The predicted levels are in good agreement with the experimental values. The pre-dose and post-dose relative abundances of N-terminal pyroglutamate approach the steady-state levels following an extended period of dosing. Each dosing time is indicated with an arrow “↑”.

The predicted pre-dose and post-dose concentrations of MAB1 with N-terminal pyroglutamate (Fig 6A, hollow circles) are consistent with the experimental results (Fig 6A, solid dots). The pre-dose and post-dose concentrations of MAB1 with N-terminal pyroglutamate will also approach the steady-state concentrations after a finite number of doses (Fig 6A, hollow circles). Using Eqs 8, 9, 16 and 17, the pre-dose and post-dose concentration of MAB1 with N-terminal pyroglutamate are 6.6 μg/mL and 7.5 μg/mL, respectively. At the 12^th^ dose (22 weeks, 154 days), the predicted pre-dose and post-dose concentrations of MAB1 with pyroglutamate are 6.5 μg/mL and 7.4 μg/mL, respectively, close to the steady-state concentrations.

### Modeling the subject exposures to the MAB1 PQAs in the multiple-dose study

The subject exposure to both total MAB1 and MAB1 possessing a specific attribute (e.g. Asn deamidation) can be calculated for any given time interval using the area under the concentration-time curves. The AUCs of the predicted and experimental total MAB1 serum concentration-time curves represent the subject exposures to total MAB1 over a defined time interval. Based on the predicted and experimental MAB1 serum concentration-time curves (Fig 5A), the predicted and experimentally determined subject exposures to total MAB1 over the course of 5 doses (56 days) were 5013 μg/mL∙day and 4789 μg/mL∙day, respectively. The difference between the predicted and experimental values is 4.7%. Similarly, the AUCs of the predicted and experimentally determined concentration-time curves of MAB1 with deamidation at Asn site 2 represent the subject exposures to MAB1 with deamidation at Asn site 2 over a defined time interval. The predicted and experimentally determined subject exposures to the Asn site 2-deamidated MAB1 over the course of 5 doses (56 days) were 381.5 μg/mL∙day and 352.6 μg/mL∙day, respectively (Fig 5A). The predicted and experimentally determined subject exposures to the Asn site 2-deamidated MAB1 as a fraction of the subject exposure to total MAB1 over 56 days were 7.0% and 7.9%, respectively, demonstrating that the models can accurately predict the experimental results.

The predicted and experimentally determined subject exposures to the Asn site 3-deamidated MAB1 over the course of 5 doses (56 days) were 172.8 μg/mL∙day and 158.8 μg/mL∙day, respectively (Fig 5A). The predicted and experimentally determined subject exposures to the Asn site 3-deamidated MAB1 as a fraction of the subject exposure to total MAB1 over the span of 56 days were 3.4% and 3.3%, respectively.

The predicted and experimentally determined subject exposures to MAB1 with N-terminal pyroglutamate over the course of 5 doses (56 days) were 218.5 μg/mL∙day and 231.8 μg/mL∙day, respectively (Fig 6A). The predicted and experimentally determined subject exposures to MAB1 with N-terminal pyroglutamate as a fraction of the subject exposure to total MAB1 over 56 days were 4.3% and 4.8%, respectively.

### Modeling the in vivo relative abundances of the MAB1 PQAs in the multiple-dose study

The models can also be used to predict the pre-dose and post-dose levels of a PQA of a mAb at any given dose during a multiple-dose study using Eqs 12 and 13, respectively. For example, the predicted pre-dose and post-dose levels of deamidation at Asn site 2 on Day 56 are 14.2% and 7.9%, respectively. The predicted values are consistent with the LC/MS determined pre-dose and post-dose on Day 56, which were 15.0% and 7.9%, respectively. All other predicted levels of deamidation (Fig 5B, hollow circles) are in good agreement with the experimental measurements (Fig 5B, solid dots). The pre-dose and post-dose PTM levels in a multiple-dose study will reach a steady state as the pre-dose and post-dose mAb serum concentrations reach the steady-state concentrations (Fig 5B). The predicted pre-dose and post-dose steady-state levels of an PQA (i.e., the plateau levels) correspond to the maximum and minimum levels of this PQA *in vivo* in a multiple-dose study. Using Eqs 16 and 17, the pre-dose and post-dose steady-state levels of MAB1 with deamidation at Asn site 2 were determined to be 17.9% and 9.7%, respectively.

Similarly, the predicted pre-dose and post-dose levels of deamidation at Asn site 3 (Fig 5B, hollow triangles) are in good agreement with the experimental measurements (Fig 5B, solid triangles). The pre-dose and post-dose steady-state levels of MAB1 with deamidation at Asn site 3 were determined to be 8.4% and 4.6%, respectively.

The predicted pre-dose and post-dose levels of N-terminal pyroglutamate (Fig 6B, hollow circles) are in good agreement with the experimental measurements (Fig 6B, solid dots). The pre-dose and post-dose steady-state levels of MAB1 with N-terminal pyroglutamate were determined to be 8.1% and 4.5%, respectively.

### Example applications of the models

**Fig 7:**
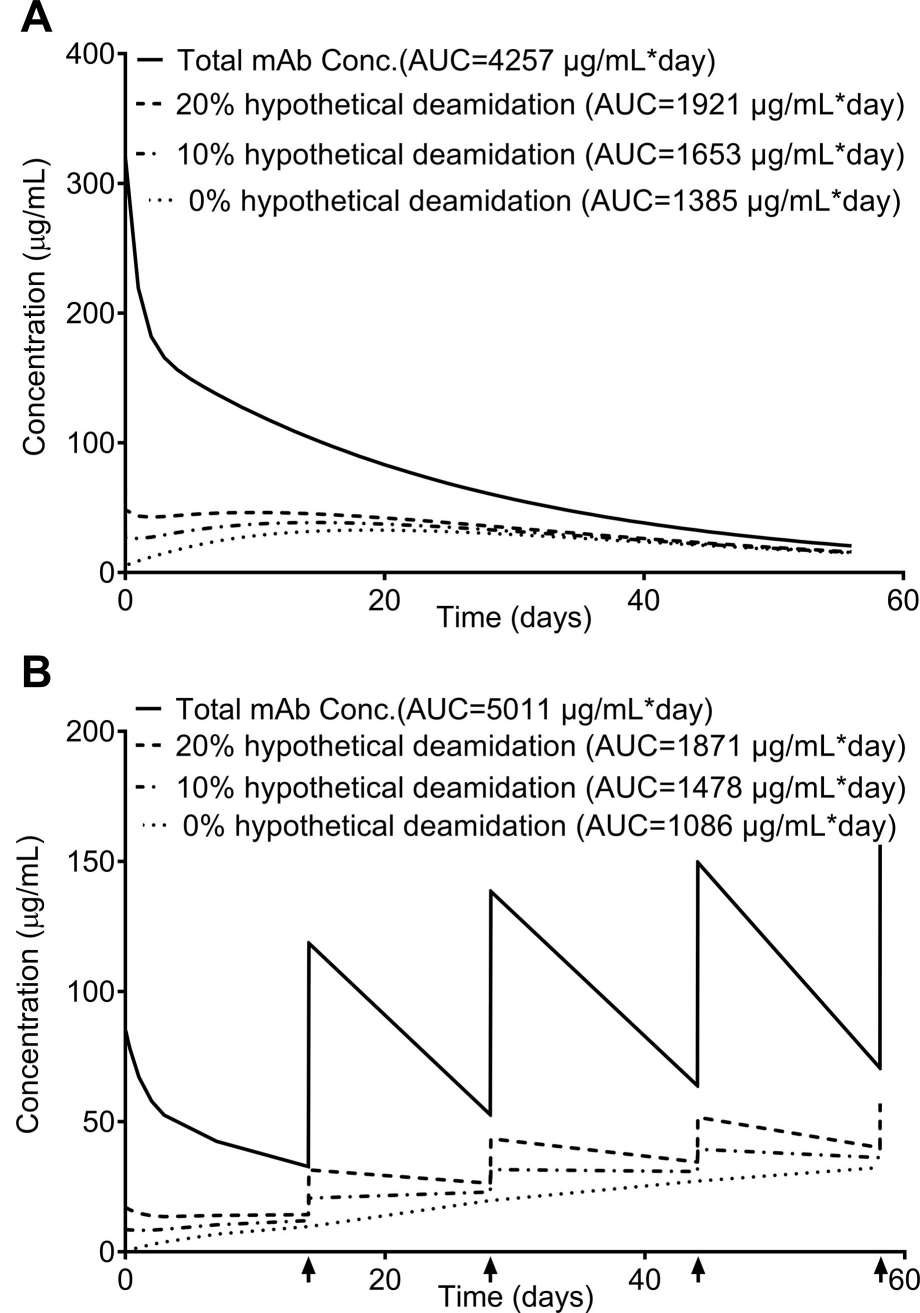
**Modeling the subject exposures to a hypothetical CDR deamidation with an *in vivo* deamidation rate of 2.5% per day^-1^ and initial deamidation levels at 0%, 10%, and 20% in the single-dose study (A) and the multiple-dose study (B)**. **(A)** The subject exposure to the hypothetical CDR deamidated variants with 0%, 10%, and 20% initial deamidation over 56 days in the single-dose study are 1385 μg/mL∙day, 1653 μg/mL∙day, and 1921 μg/mL∙day, respectively, consisting of 32.5%, 38.8%, and 45.1% subject exposure to the total mAb, respectively. **(B)** The subject exposure to the hypothetical CDR deamidated variants with 0%, 10%, and 20% initial deamidation over 5 doses (56 days) in the multiple-dose study are 1086 μg/mL∙day, 1478 μg/mL∙day, and 1871 μg/mL∙day, respectively, consisting of 21.7%, 29.5%, and 37.3% subject exposure to the total mAb, respectively Each dosing time is indicated with an arrow “↑”.

Quantitative assessment of PQAs of a therapeutic protein *in vivo* is important because it helps to identify potential critical quality attributes (CQAs) that would not otherwise be identified *in vitro*, thereby influencing product risk assessment and control strategy. The models described in this paper can be used to quantitatively assess of PQAs in many applications. First, the *in vivo* progression of PQAs and the subject exposures to the PQAs in single and multiple-dose regimens can be calculated using these models. The good agreements between model predictions and experimental measurements have been demonstrated in the sections above (Figs 4–6). Second, the models can be used to predict the subject exposure by extending the dosing period (*t*) and determine the pre-dose and post-dose steady-state concentrations/levels of PQAs (Figs 5 and 6), providing insights to pre-clinical and clinical studies. Third, the models can be used to evaluate the impact of changes in the initial levels of PQAs resulting from process changes or lot-to-lot variability by adjusting the initial levels of PQAs (*P*_0_) in the modeling equations. This is particularly meaningful if the PQA is a CDR modification. Since the MAB1 used in this work does not have a CDR modification suitable for this modeling application, we used a hypothetical CDR deamidation with an *in vivo* deamidation rate of 2.5% per day^-1^ as an example of using the models to assess the impact of the initial levels of CDR deamidation on the subject exposure to PQAs. If there were three batches of drug products with the initial levels of the hypothetical CDR deamidation of 0%, 10%, and 20%, respectively. The hypothetical CDR deamidation *in vivo* overtime of these batches can be described as *P*_*deam*_(*t*) = 1−(1−0)∙*e*^−0.025*t*^, *P*_*deam*_(*t*) = 1−(1−0.1)∙*e*^−0.025*t*^, and *P*_*deam*_(*t*) = 1−(1−0.2)∙*e*^−0.025*t*^, respectively. Using the models described above, the subject exposure to the hypothetical CDR deamidated variants with 0%, 10%, and 20% initial deamidation over 56 days in the single-dose study are 1385 μg/mL∙day, 1653 μg/mL∙day, and 1921 μg/mL∙day, respectively, consisting of 32.5%, 38.8%, and 45.1% subject exposure to the total mAb, respectively (Fig 7A). For multiple-dose study, the subject exposure to the hypothetical CDR deamidated variants with 0%, 10%, and 20% initial deamidation over 5 doses (56 days) are 1086 μg/mL∙day, 1478 μg/mL∙day, and 1871 μg/mL∙day, respectively, consisting of 21.7%, 29.5%, and 37.3% subject exposure to the total mAb, respectively (Fig 7B). Thus, if the process control resulted in ±10% of initial levels of this hypothetical CDR deamidation, the subject exposure to this CDR deamidated mAb varies about 6–8% in the single- or multiple-dose studies over 56 days. Since the modeling demonstrates that the subject exposure to this hypothetical CDR deamidation is insensitive to the initial level of the PQA, the acceptable ranges of the PQA could be widened to allow justification of process risk assessment. Combining with the effect of a CDR modification on the potency measured by a potency assay, the modeling results can be used to estimate the subject exposure to effective drug when the initial CDR modification changes resulted from process controls or lot-to-lot variation, providing critical information for product risk assessment.

## Discussion

The dynamic *in vivo* environment of the circulating bloodstream, resulting in mAb clearance, elimination, and degradation, is much more relevant for assessing changes of PQAs in patients compared to the static incubation of mAbs in PBS or serum *in vitro*[10]. Thus, *in vivo* PQA quantitation and modeling provide critical information, which would not obtain from the *in vitro* studies, to assess the criticality of the PQAs. For example, our results (S11 Fig) and previous studies[11, 19] have shown that *in vivo* C-terminal lysine is rapidly removed from an antibody within one day. Similarly, trisulfide bonds have been shown to be rapidly converted to disulfide bonds *in vivo*[11]. Our results (S9 Fig) and previous studies[11, 12] demonstrated that the levels of methionine oxidation are often unaffected following *in vivo* administration. Therefore, C-terminal lysine removal, trisulfide bond, and methionine oxidation are less likely to be considered CQAs. In contrast, deamidation were shown to accumulate *in vivo* in single- and multiple-dosing regimens (Fig 3) and previous studies[11, 12]. Deamidation that occurs and accumulates at the CDR region could potentially impact on drug efficacy[30, 31] or cause off-target binding. Thus, modeling CDR deamidation *in vivo* using the equations described in this paper can quantitatively assess the impact of the CDR deamidation on the subject exposures, as we demostrated above (Fig 7).

In this work, asparagine deamidation and N-terminal pyroglutamate formation were used as representative PTMs to demonstrate the validity of our models. Our models can also be applied to other PTMs that exhibit either increasing or decreasing trends. For example, we applied the models to assess Mannose 5 clearance by adopting the third-order kinetic because of the enzymatic based clearance of Mannose 5. The predicted values matched well with the experimental data even though the relative abundance of Mannose 5 decreased to below 1.0% over time (S14 Fig). Our models can be used to calculate other parameters to evaluate a given attribute or PTM in a multiple-dose study. These parameters include the accumulation rate to the steady state, the average level at the steady state, and the degree of variability of a PTM at the steady state.

The relative abundance of PTM was calculated using the ratio of peak area of the modified peptides to the sum of modified and native peptides. The difference in electrospray ionization efficiency of modified and native peptides may slightly impact on the accuracy of the relative quantification of PTMs. However, it does not impact on monitor the relative change of PTM *in vivo* over time. Spiking in known amount of heavy isotopically labelled native and modified peptides into samples could improve the quantification accuracy but it is difficult to obtain heavy isotopically labelled modified peptides.

The models presented in this paper is to provide bioanalytical chemists a simplified modeling tool to assess PQAs *in vivo* when advanced PK models is not available, and by no means to replace the advanced PK models. As a simplified modeling tool, there are some factors and assumptions needs to be considered when applications of these models were attempted. First, the models can only be used to predict individual PQAs, and are not suitable for the prediction of a combination of multiple PQAs. Second, the MAB1 serum concentration modeling in the multiple-dose PK study assumed that the mAb serum concentrations accumulated linearly obeying the superposition principle. All parameters of the modeling equations were determined from a single-dose study or the first dose interval from a multiple-dose study. We also simplified the calculation of the concentration-time curves and the corresponding AUCs by using only the pre-dose and post-dose values for all subsequent doses after the initial dose, simplifying the decline of the concentration during the dosing interval as linear decrease. Although the modeling calculations are consistent with the experimental results to accurately predict the PTM progression and the subject exposure to mAb PTM variants within a given limited time interval, non-linearity was observed when the experimental results were used to fit to the concentration-time equation for a longer time period (>72 days, S15 Fig) in the single-dose PK study, probably due the targeted-mediated drug disposition. The observed non-linearity suggests that time-dependent factors can impact the fitting parameters (e.g., *α* in Table 2) and therefore should be considered for long extended dosings. However, for the prediction of PTM maxima and minima from infinity m dose in the multiple-dose study, the contribution of the targeted-mediated drug disposition is negligible in that the dose amount for therapeutic antibody is usually high enough and the dosing interval is typically short enough.

## Conclusions

In summary, we quantitatively assess common PTMs of a therapeutic antibody (MAB1), and modeled the *in vivo* behaviors of PTMs for both single- and multiple-dose regimens to evaluate the impact of *in vivo* PTMs. Three asparagine residues located in the Fc region of MAB1 exhibited different deamidation rates. The levels of oxidation at three methionine residues in the Fc region of MAB1 showed no change over time *in vivo*. N-terminal pyroglutamate formed rapidly *in vivo*. C-terminal lysine was completely removed within one day. MAB1 possessing high mannose revealed the accelerated clearance. We used two Asn deamidation and the N-terminal pyroglutamate formation as representative PQAs and built modeling equations to calculate the serum concentrations of PQAs, the subject exposures to PQAs, and the *in vivo* relative abundances of PQAs both single- and multiple-dose regimens. The model predictions were validated by the experimental measurements. Thus, the models can be used to simulate the *in vivo* PQA progression and the subject exposure to PQAs in single- and multiple-dose regimens, providing quantitative approaches for the criticality assessment of PQAs of therapeutic mAbs.

## Supporting information

S1 Fig MS/MS spectra of Met site 2 containing peptide. The MS/MS spectrum of the modified peptide (top panel) and the MS/MS spectrum of wild-type peptide (bottom panel).
(TIF)

S2 Fig MS/MS spectra of Met site 3 containing peptide. The MS/MS spectrum of the modified peptide (top panel) and the MS/MS spectrum of wild-type peptide (bottom panel).
(TIF)

S3 Fig MS/MS spectra of Asn site 1 containing peptide. The MS/MS spectrum of the modified peptide (top panel) and the MS/MS spectrum of wild-type peptide (bottom panel).
(TIF)

S4 Fig MS/MS spectra of Asn site 2 containing peptide. The MS/MS spectrum of the modified peptide (top panel) and the MS/MS spectrum of wild-type peptide (bottom panel).
(TIF)

S5 Fig MS/MS spectra of Asn site 3 containing peptide. The MS/MS spectrum of the modified peptide (top panel) and the MS/MS spectrum of wild-type peptide (bottom panel).
(TIF)

S6 Fig MS/MS spectra of heavy chain N-terminal peptide. The MS/MS spectrum of the N-terminal pyroglutamate peptide (top panel) and the MS/MS spectrum of wild-type peptide (bottom panel).
(TIF)

S7 Fig MS/MS spectra of heavy chain C-terminal peptide. The MS/MS spectrum of the C-terminal peptide with lysine (top panel) and the MS/MS spectrum of the C-terminal peptide without lysine (bottom panel).
(TIF)

S8 Fig MS/MS spectra of mannose 5 containing peptide. The MS/MS spectrum of the mannose 5 containing peptide (top panel) and the MS/MS spectrum of the wild-type peptide (bottom panel).
(TIF)

S9 Fig **The relative abundance of oxidation at each of the three Met sites in the MAB1 Fc regions from the single-dose PK study (A) and the multiple-dose PK study (B)**. **(A)** In the single-dose study, the relative abundance of Met oxidation fluctuated slightly but remained nearly unchanged at all three sites. **(B)** In the multiple-dose study, the relative abundance of oxidation at Met site 1 decreased slightly during each dosing interval and increased slightly after each dose. The relative abundances of oxidation at Met site 2 and 3 remained stable. Each dosing time is indicated with an arrow “↑”.
(TIF)

S10 Fig **The relative abundances of N-terminal pyroglutamate from the single-dose PK study (A) and the multiple-dose PK study (B)**. **(A)** In the single-dose study, the relative abundance of N-terminal pyroglutamate increased over time. **(B)** In the multiple-dose study, the relative abundance of N-terminal pyroglutamate increased during each dosing interval but decreased sharply following each subsequent dose of MAB1 due to dilution with newly administrated unmodified MAB1, exhibiting an upward trending saw-tooth pattern. Each dosing time is indicated with an arrow “↑”.
(TIF)

S11 Fig **The relative abundances of MAB1 possessing a heavy chain C-terminal lysine from the single-dose PK study (A) and the multiple-dose PK study (B)**. In both single and multiple-dose studies, the C-terminal lysine was rapidly removed within one day following each dose. Each dosing time is indicated with an arrow “↑”.
(TIF)

S12 Fig **The relative abundances of Mannose 5 glycoform from the single-dose PK study (A) and the multiple-dose PK study (B)**. **(A)** In the single-dose study, the relative abundance of Mannose 5 decreased from 0.5% to undetectable within 6 weeks. **(B)** In the multiple-dose study, the relative abundance of Mannose 5 decreased during each dosing interval but sharply increased at each subsequent new dose because of newly administrated MAB1 with a higher level of Mannose 5, exhibiting a downward trending saw-tooth pattern. Each dosing time is indicated with an arrow “↑”.
(TIF)

S13 Fig The relative abundances of major glycoforms from the single-dose PK study. (TIF)

S14 Fig Model predictions and experiment measurements of mannose 5 clearance from the single-dose PK study. (TIF)

S15 Fig Model predictions and experiment measurements of the serum concentration of MAB1 over 72 days. (TIF)

S1 File NC3Rs ARRIVE guidelines checklist filled. (PDF)
