# Supplementary figures and images for: Quantitation and modeling of post-translational modifications in a therapeutic monoclonal antibody from single- and multiple-dose monkey pharmacokinetic studies using mass spectrometry
Source: PLoS One. 2019 Oct 16;14(10):e0223899. doi: 10.1371/journal.pone.0223899 (PMC6795451; doi:10.1371/journal.pone.0223899)

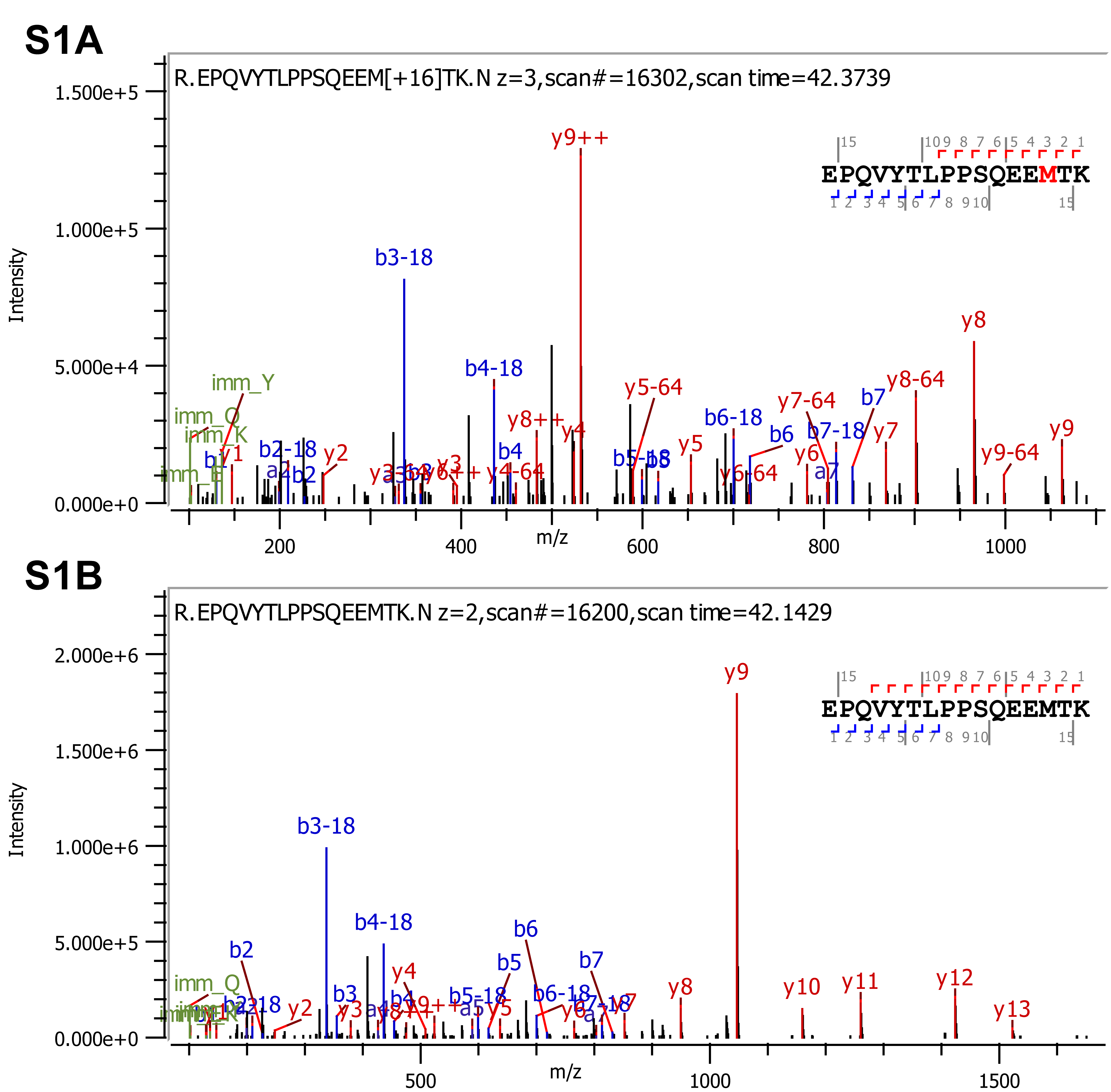

Supplement: S1 Fig — The MS/MS spectrum of the modified peptide (top panel) and the MS/MS spectrum of wild-type peptide (bottom panel). (TIF) [file pone.0223899.s001.tif]

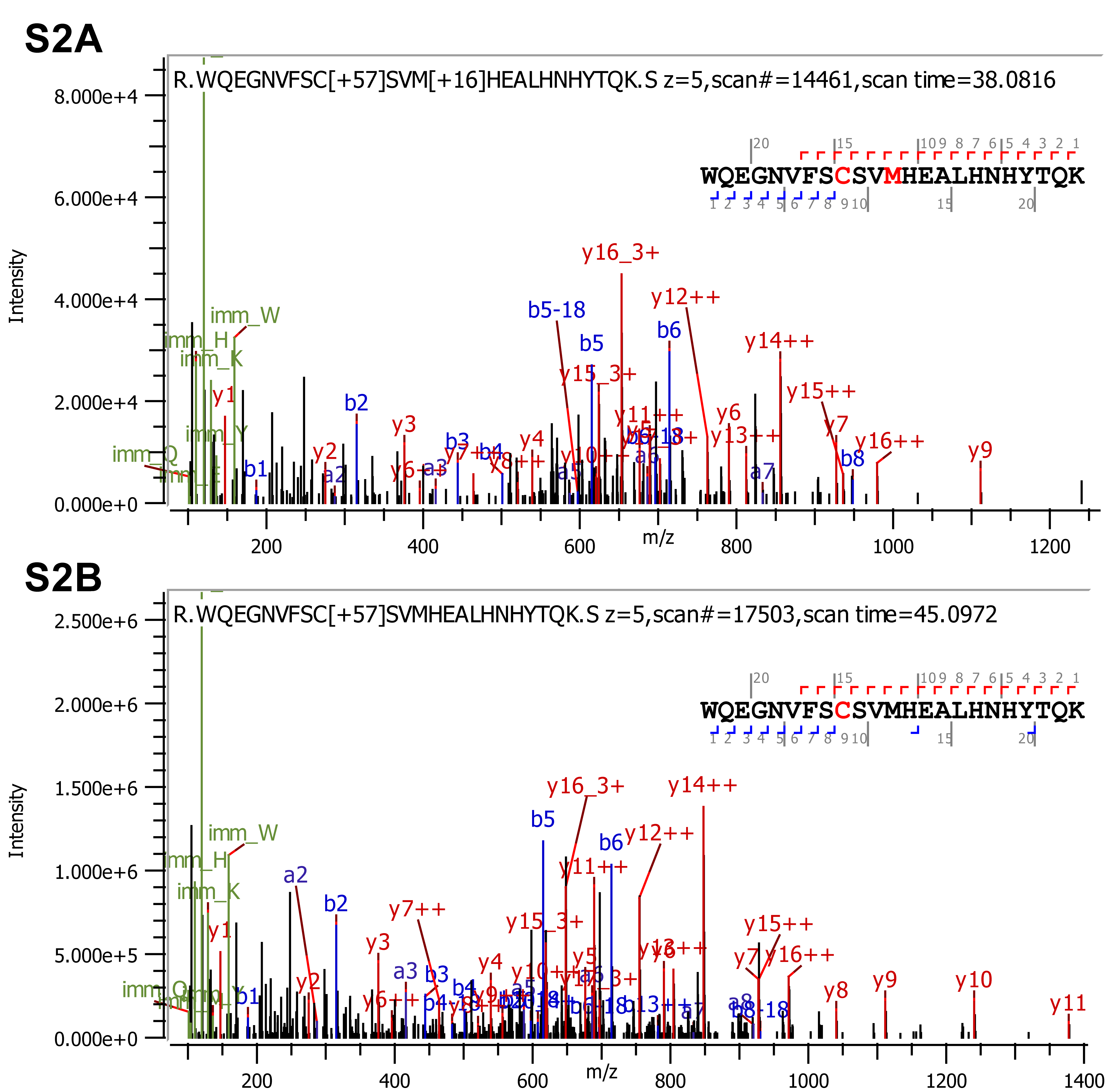

Supplement: S2 Fig — The MS/MS spectrum of the modified peptide (top panel) and the MS/MS spectrum of wild-type peptide (bottom panel). (TIF) [file pone.0223899.s002.tif]

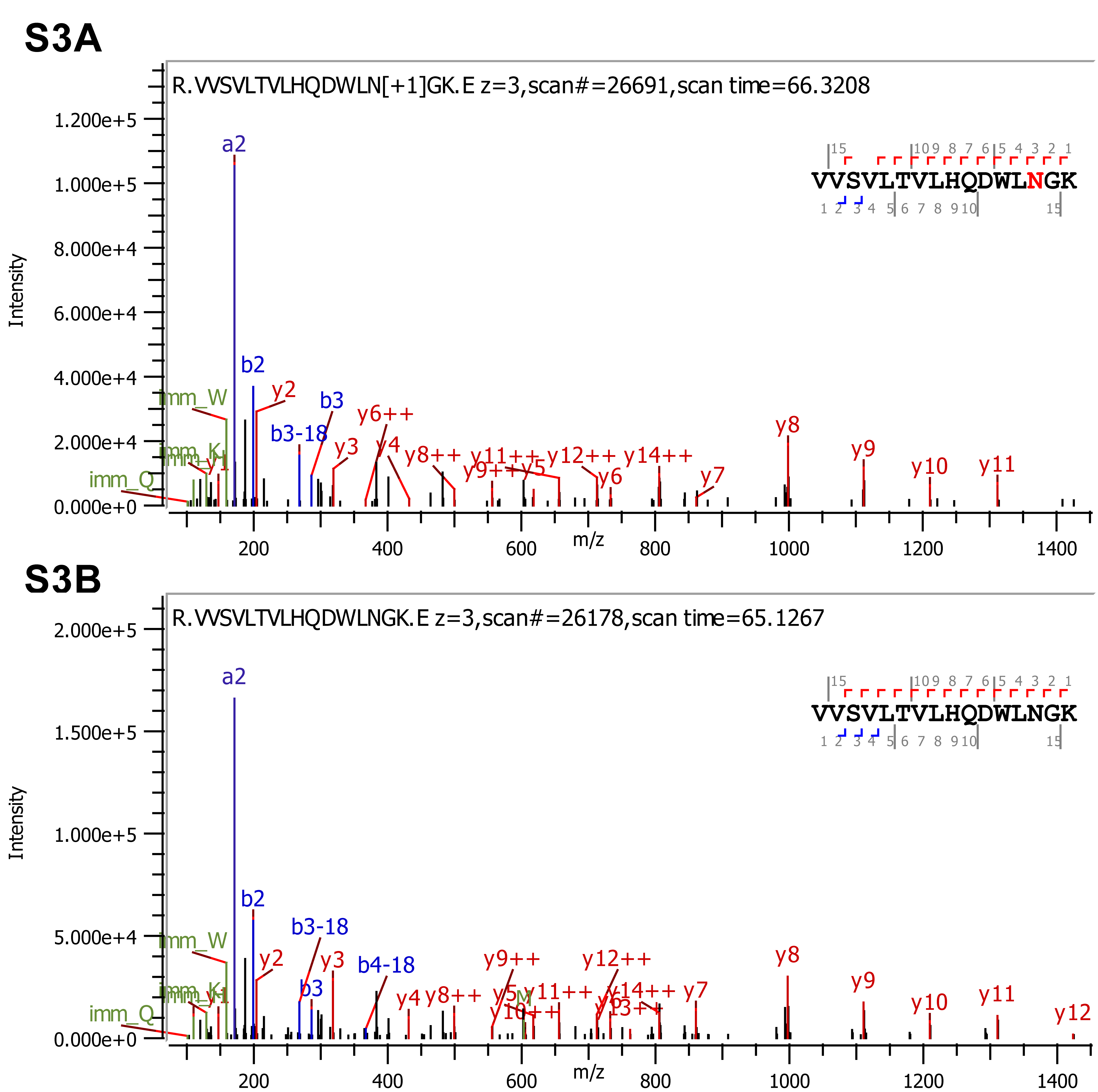

Supplement: S3 Fig — The MS/MS spectrum of the modified peptide (top panel) and the MS/MS spectrum of wild-type peptide (bottom panel). (TIF) [file pone.0223899.s003.tif]

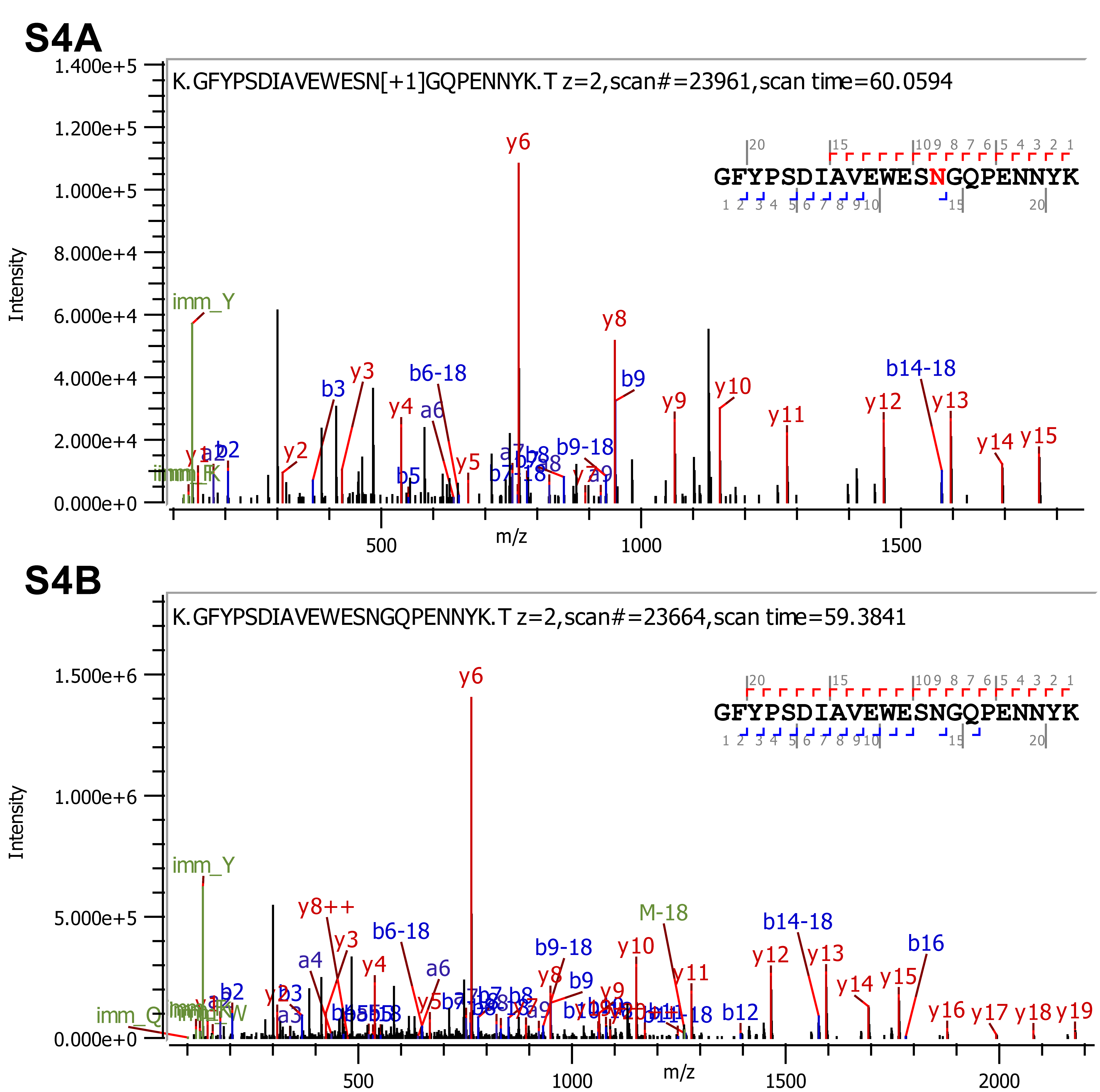

Supplement: S4 Fig — The MS/MS spectrum of the modified peptide (top panel) and the MS/MS spectrum of wild-type peptide (bottom panel). (TIF) [file pone.0223899.s004.tif]

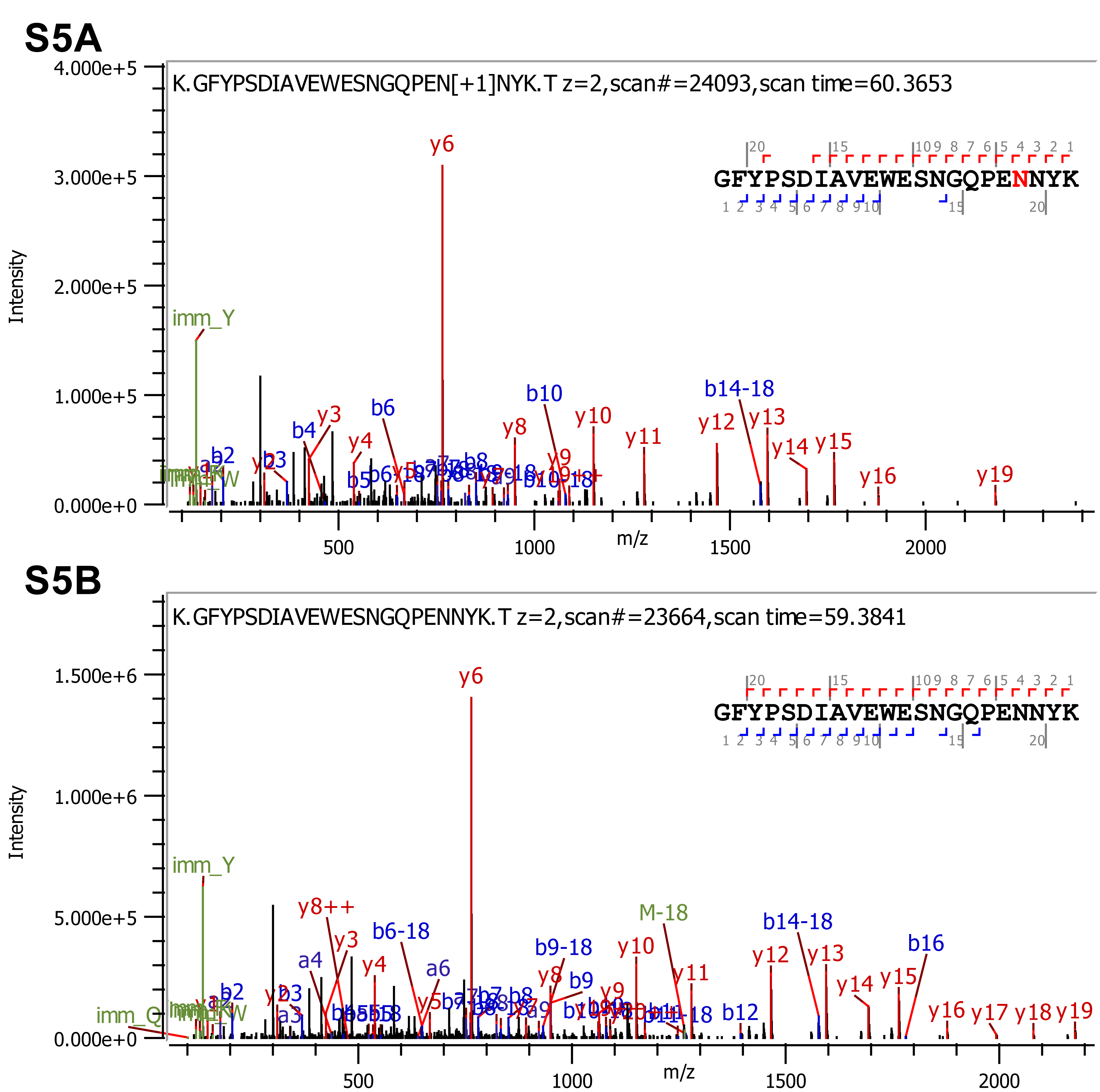

Supplement: S5 Fig — The MS/MS spectrum of the modified peptide (top panel) and the MS/MS spectrum of wild-type peptide (bottom panel). (TIF) [file pone.0223899.s005.tif]

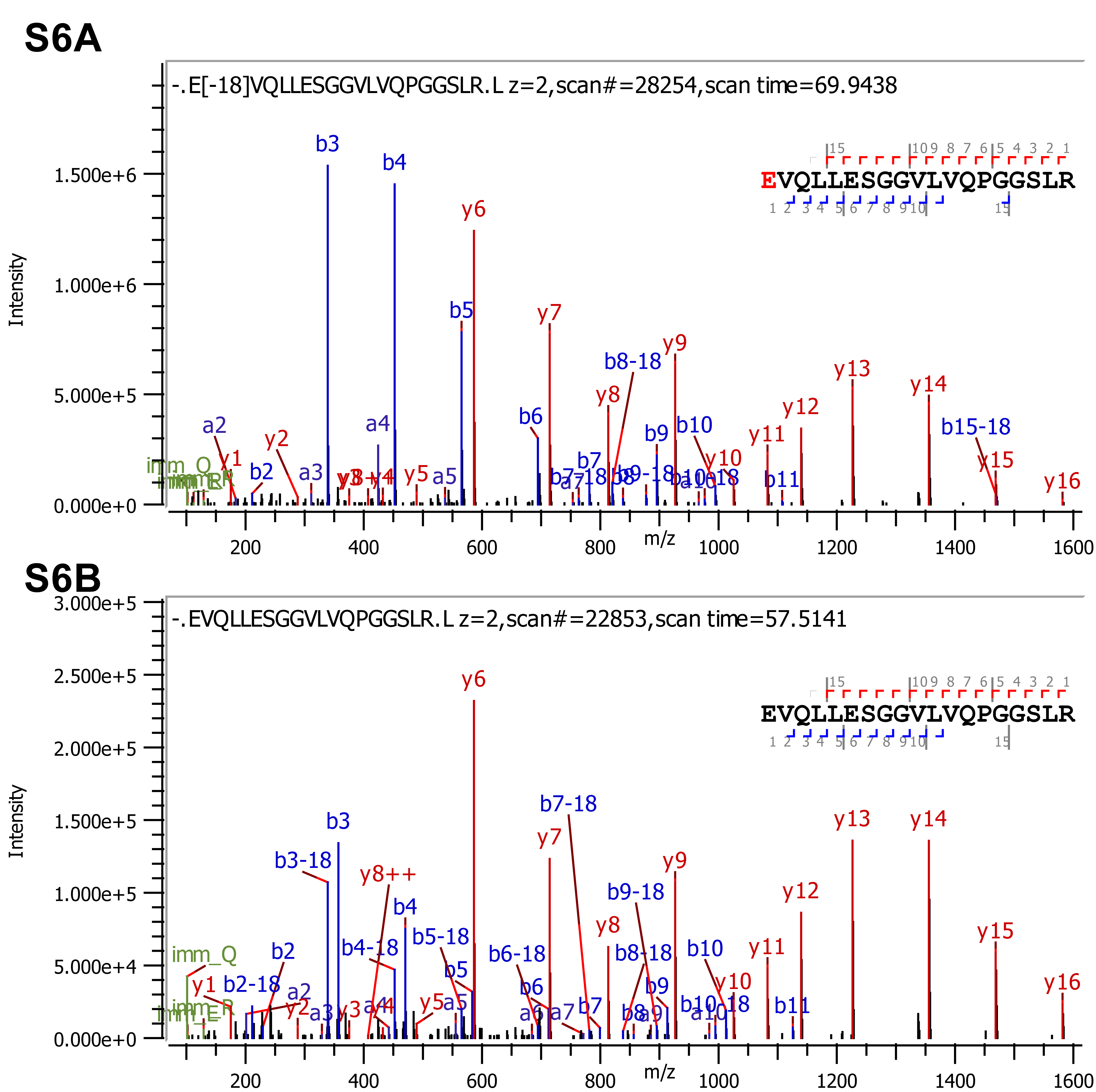

Supplement: S6 Fig — The MS/MS spectrum of the N-terminal pyroglutamate peptide (top panel) and the MS/MS spectrum of wild-type peptide (bottom panel). (TIF) [file pone.0223899.s006.tif]

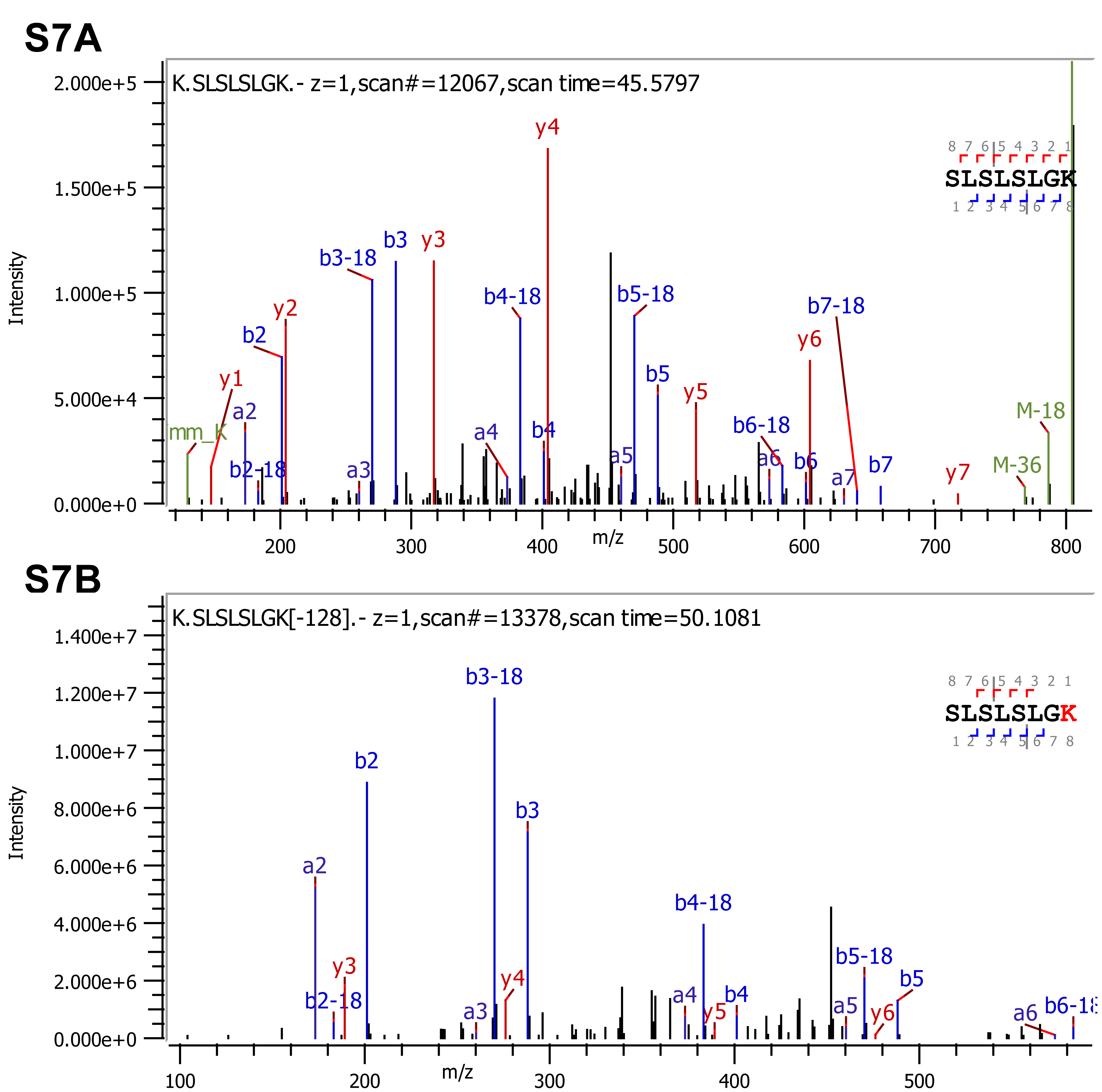

Supplement: S7 Fig — The MS/MS spectrum of the C-terminal peptide with lysine (top panel) and the MS/MS spectrum of the C-terminal peptide without lysine (bottom panel). (TIF) [file pone.0223899.s007.tif]

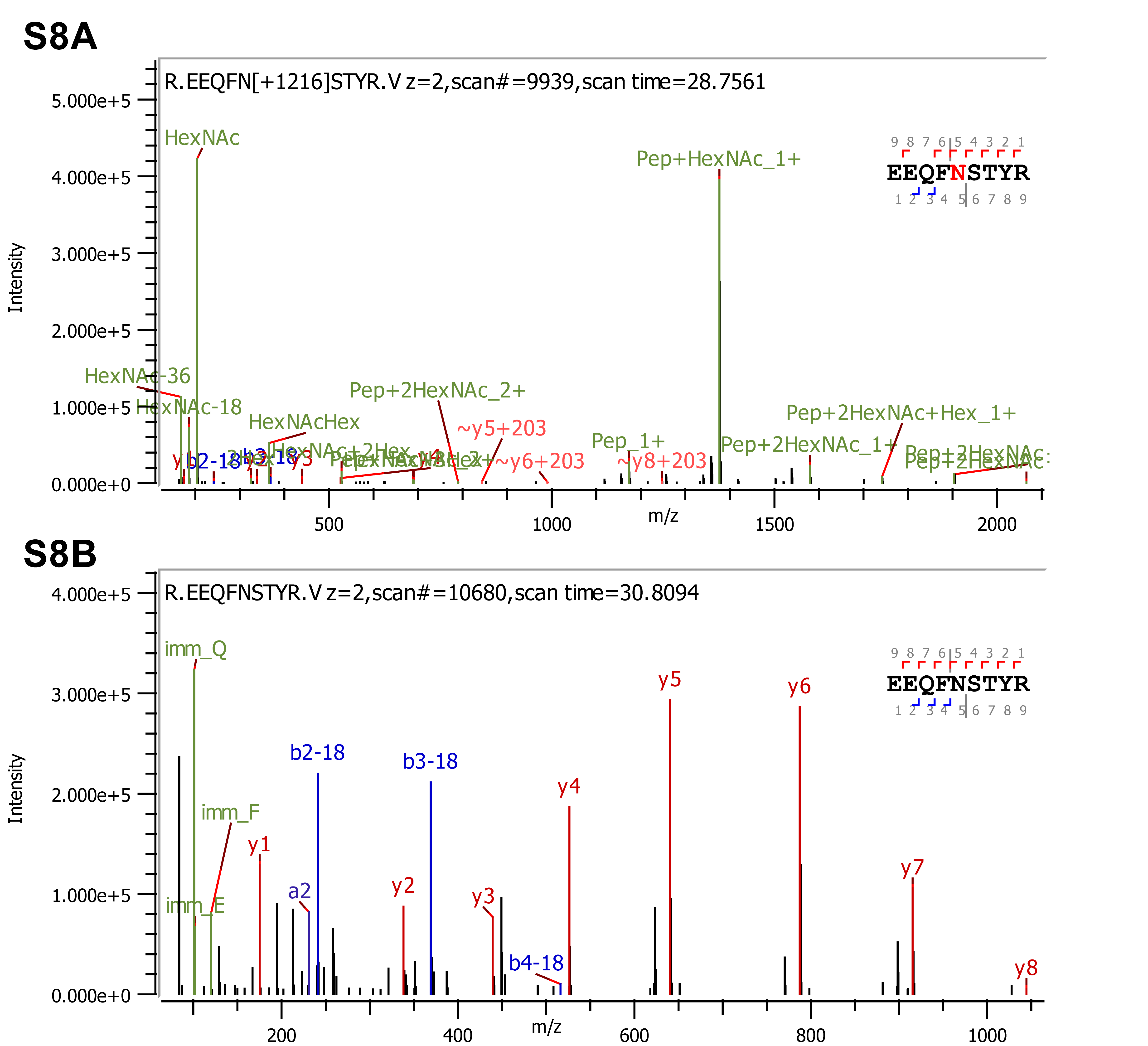

Supplement: S8 Fig — The MS/MS spectrum of the mannose 5 containing peptide (top panel) and the MS/MS spectrum of the wild-type peptide (bottom panel). (TIF) [file pone.0223899.s008.tif]

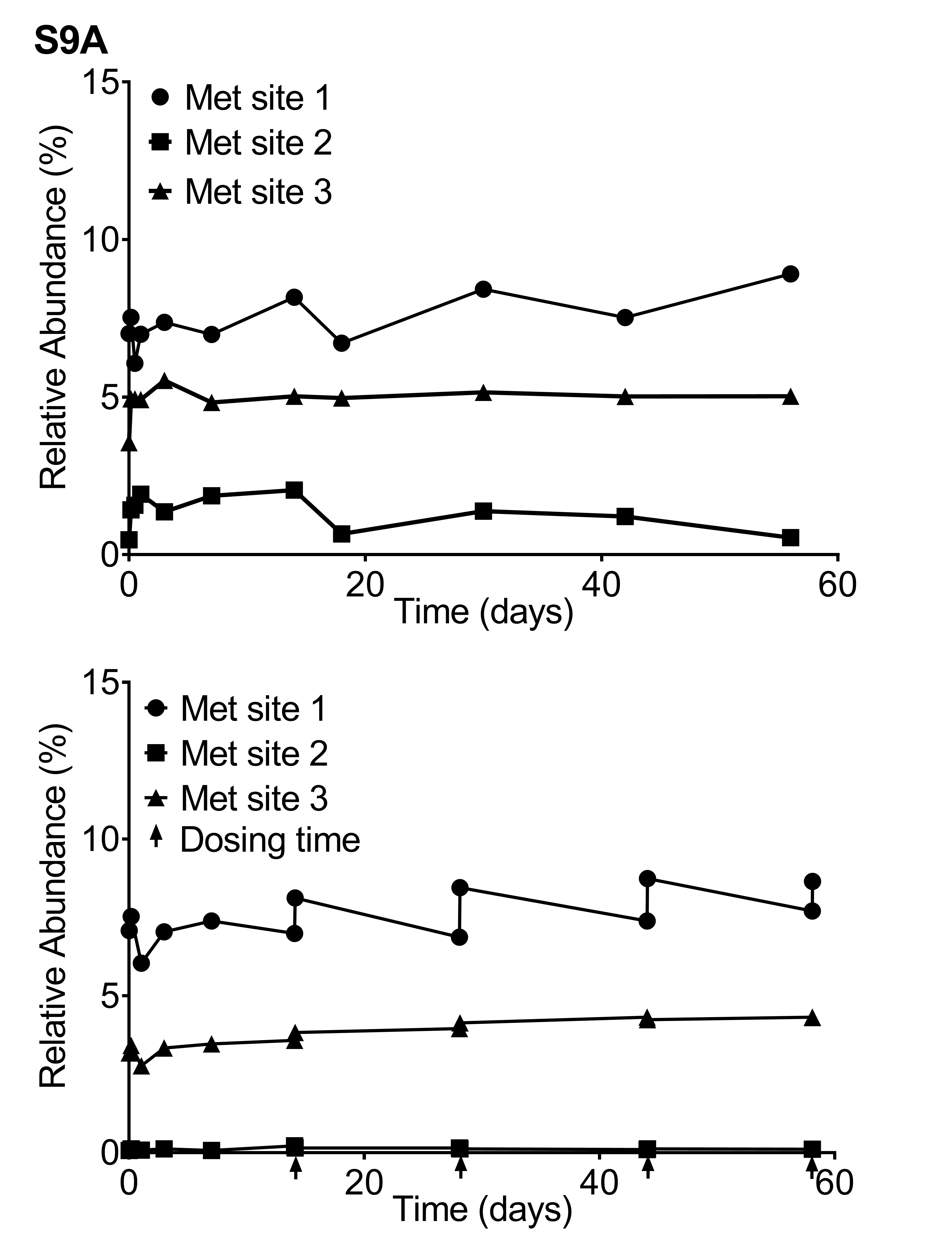

Supplement: S9 Fig — The relative abundance of oxidation at each of the three Met sites in the MAB1 Fc regions from the single-dose PK study (A) and the multiple-dose PK study (B). (A) In the single-dose study, the relative abundance of Met oxidation fluctuated slightly but remained nearly unchanged at all three sites. (B) In the multiple-dose study, the relative abundance of oxidation at Met site 1 decreased slightly during each dosing interval and increased slightly after each dose. The relative abundances of oxidation at Met site 2 and 3 remained stable. Each dosing time is indicated with an arrow “↑”. (TIF) [file pone.0223899.s009.tif]

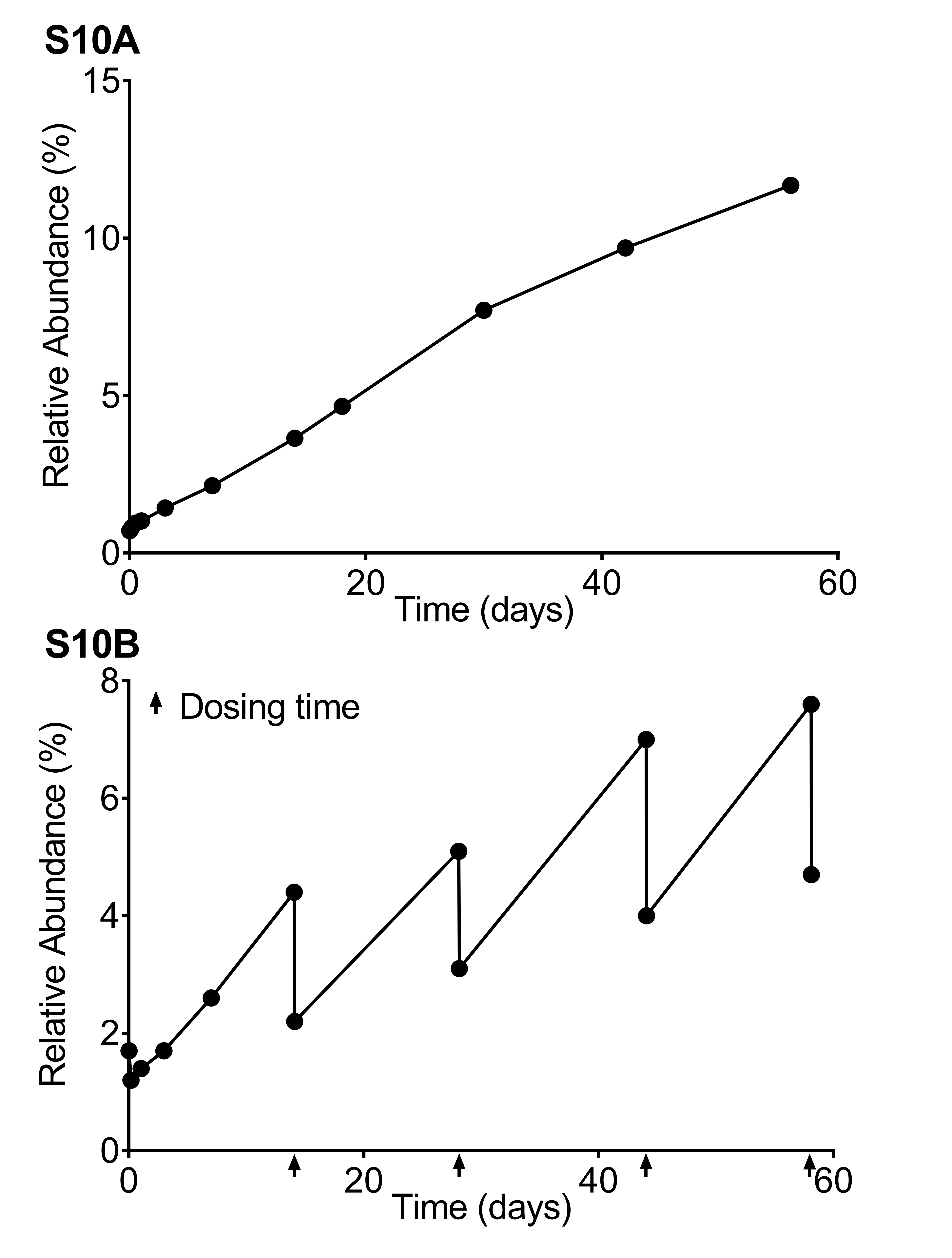

Supplement: S10 Fig — The relative abundances of N-terminal pyroglutamate from the single-dose PK study (A) and the multiple-dose PK study (B). (A) In the single-dose study, the relative abundance of N-terminal pyroglutamate increased over time. (B) In the multiple-dose study, the relative abundance of N-terminal pyroglutamate increased during each dosing interval but decreased sharply following each subsequent dose of MAB1 due to dilution with newly administrated unmodified MAB1, exhibiting an upward trending saw-tooth pattern. Each dosing time is indicated with an arrow “↑”. (TIF) [file pone.0223899.s010.tif]

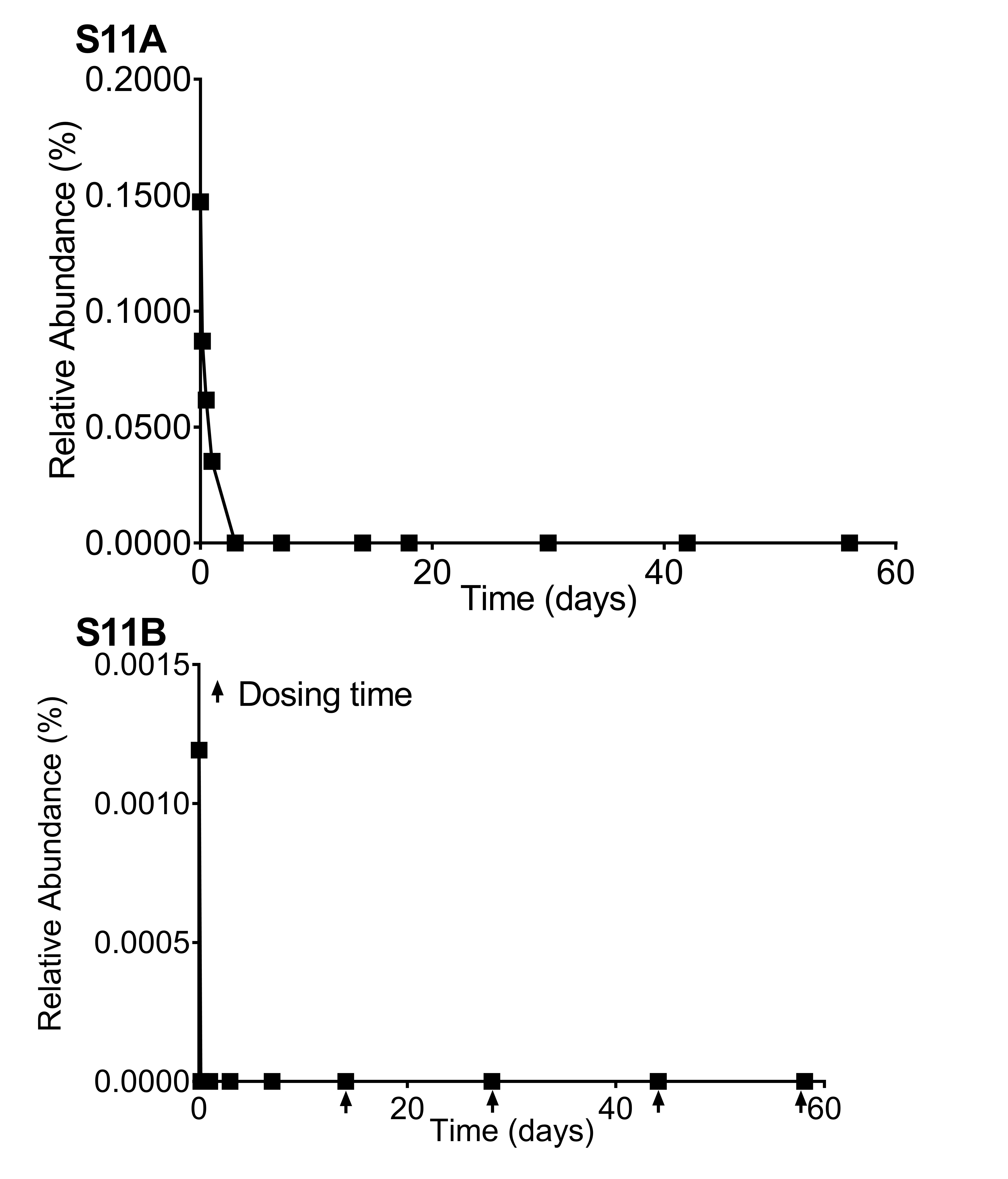

Supplement: S11 Fig — The relative abundances of MAB1 possessing a heavy chain C-terminal lysine from the single-dose PK study (A) and the multiple-dose PK study (B). In both single and multiple-dose studies, the C-terminal lysine was rapidly removed within one day following each dose. Each dosing time is indicated with an arrow “↑”. (TIF) [file pone.0223899.s011.tif]

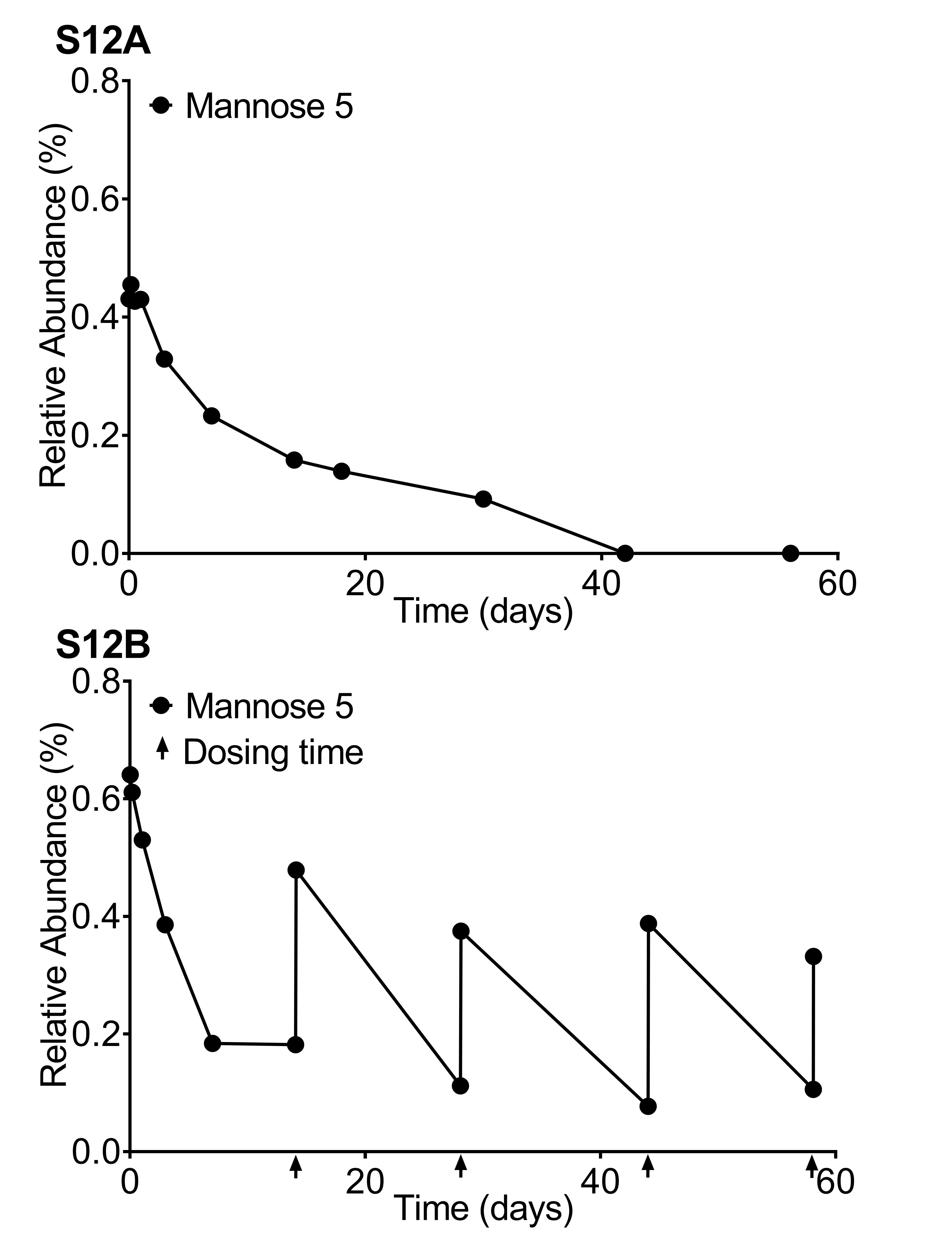

Supplement: S12 Fig — The relative abundances of Mannose 5 glycoform from the single-dose PK study (A) and the multiple-dose PK study (B). (A) In the single-dose study, the relative abundance of Mannose 5 decreased from 0.5% to undetectable within 6 weeks. (B) In the multiple-dose study, the relative abundance of Mannose 5 decreased during each dosing interval but sharply increased at each subsequent new dose because of newly administrated MAB1 with a higher level of Mannose 5, exhibiting a downward trending saw-tooth pattern. Each dosing time is indicated with an arrow “↑”. (TIF) [file pone.0223899.s012.tif]

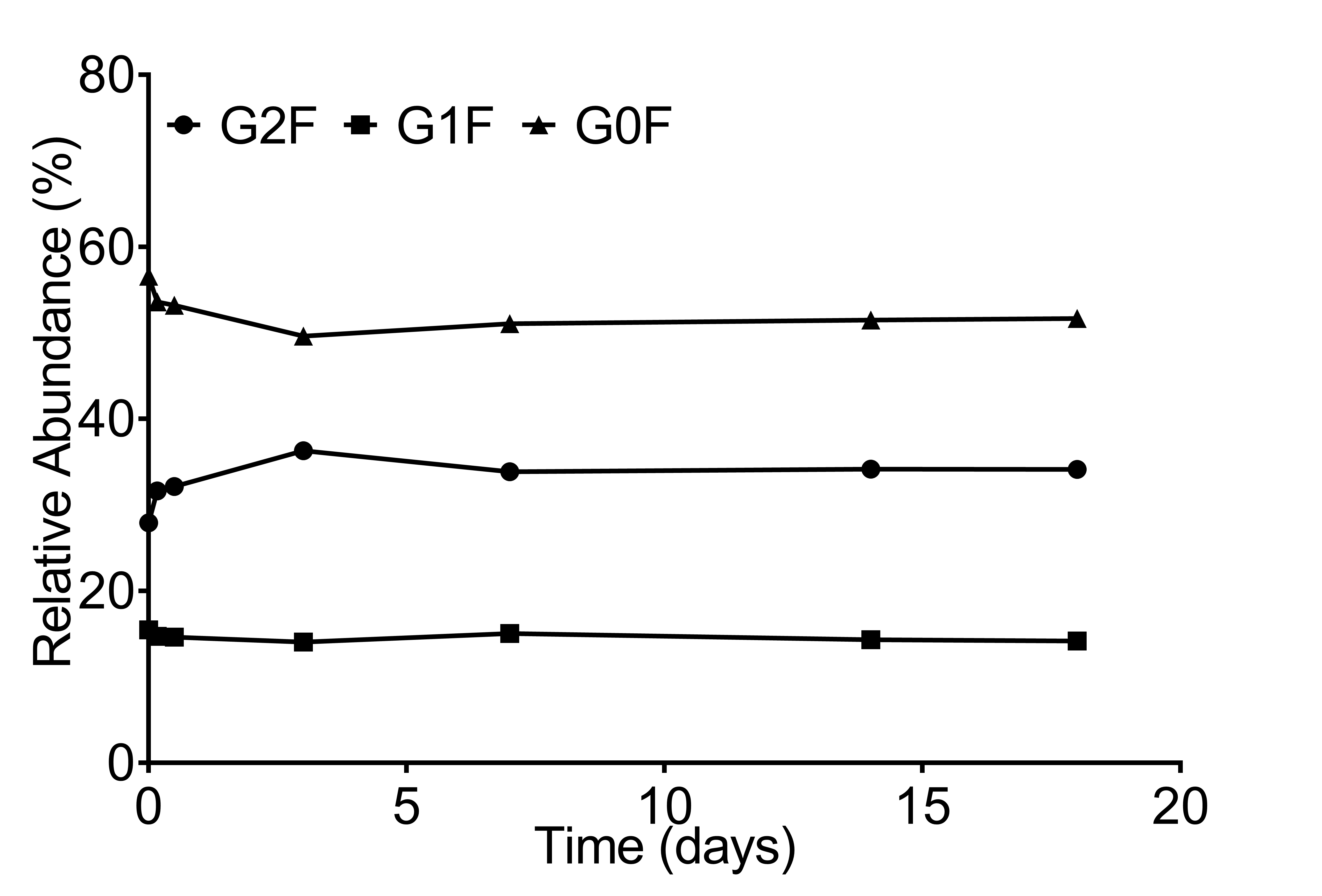

Supplement: S13 Fig — (TIF) [file pone.0223899.s013.tif]

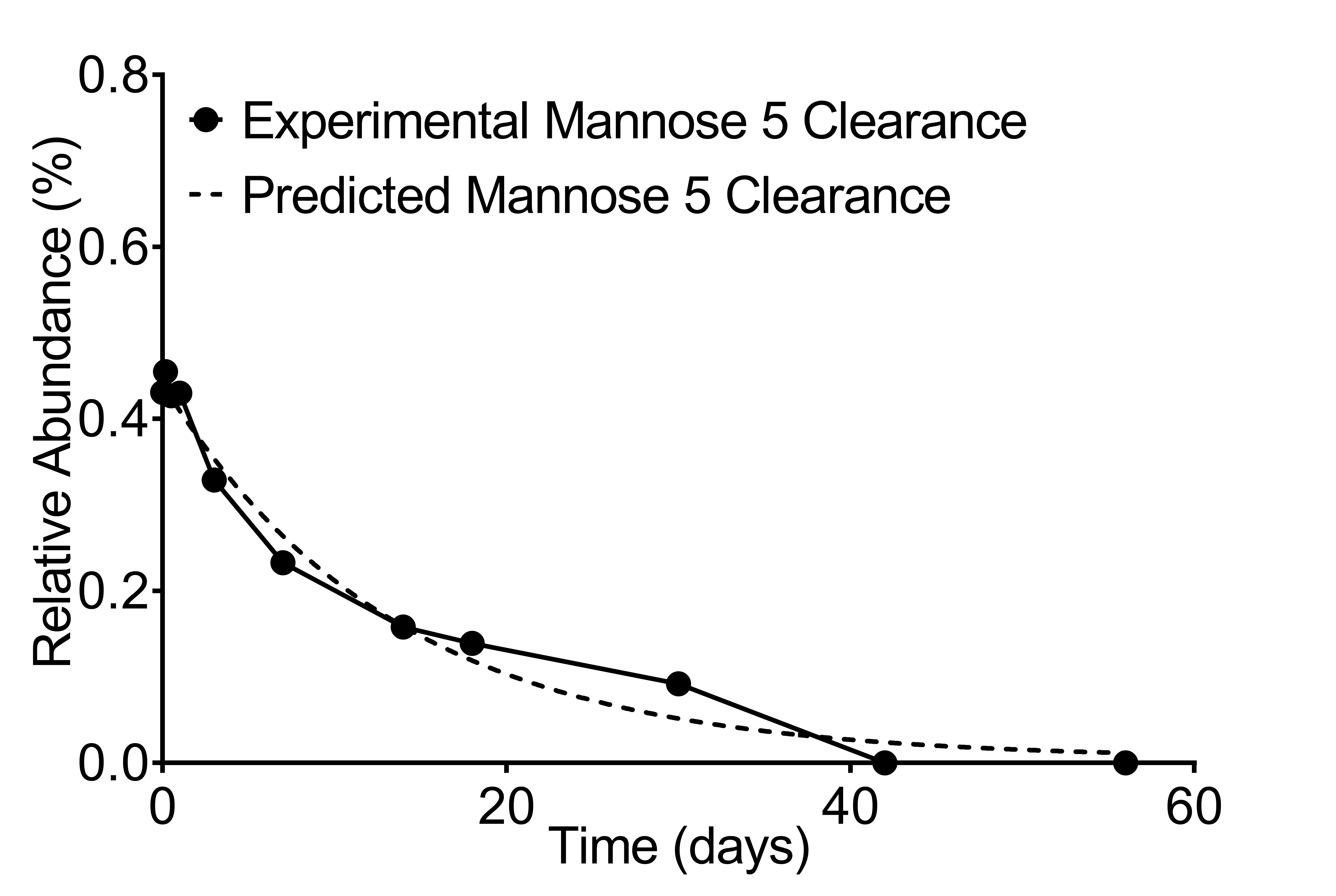

Supplement: S14 Fig — (TIF) [file pone.0223899.s014.tif]

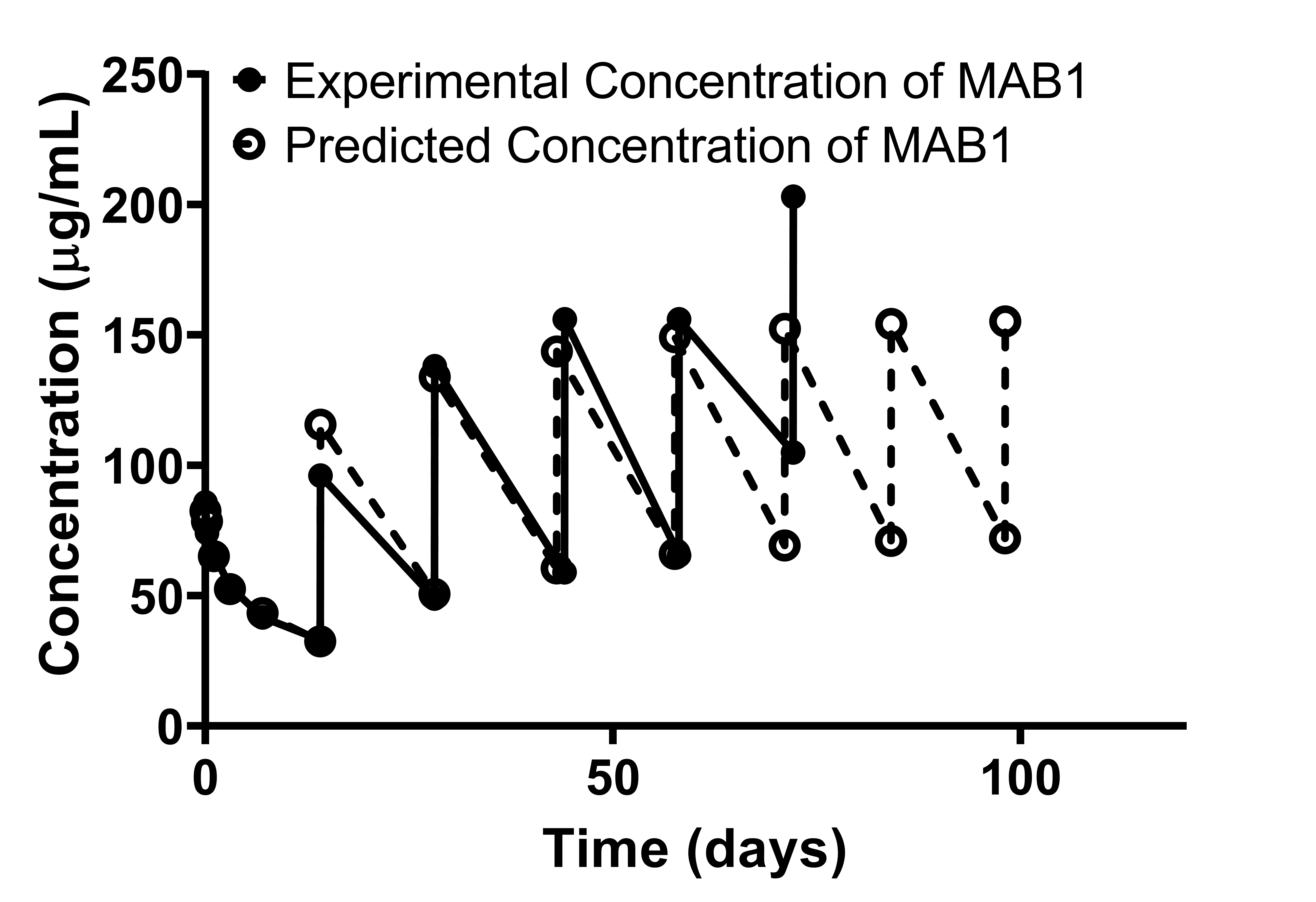

Supplement: S15 Fig — (TIF) [file pone.0223899.s015.tif]
